# Noncoding dsRNA induces retinoic acid synthesis to stimulate hair follicle regeneration via TLR3

**DOI:** 10.1038/s41467-019-10811-y

**Published:** 2019-06-26

**Authors:** Dongwon Kim, Ruosi Chen, Mary Sheu, Noori Kim, Sooah Kim, Nasif Islam, Eric M. Wier, Gaofeng Wang, Ang Li, Angela Park, Wooyang Son, Benjamin Evans, Victoria Yu, Vicky P. Prizmic, Eugene Oh, Zixiao Wang, Jianshi Yu, Weiliang Huang, Nathan K. Archer, Zhiqi Hu, Nashay Clemetson, Amanda M. Nelson, Anna Chien, Ginette A. Okoye, Lloyd S. Miller, Gabriel Ghiaur, Sewon Kang, Jace W. Jones, Maureen A. Kane, Luis A. Garza

**Affiliations:** 1 Department of Dermatology, Johns Hopkins University School of Medicine, Baltimore, MD 21287 USA; 2 Department of Plastic and Aesthetic Surgery, Nanfang Hospital of Southern Medical University, Guangzhou, 510515 Guangdong Province China; 3 Department of Pharmaceutical Sciences, School of Pharmacy, University of Maryland, Baltimore, MD 21201 USA; 4 Department of Dermatology, College of Medicine, The Pennsylvania State University, Hershey, PA 17033 USA; 5 Sidney Kimmel Cancer Center at Johns Hopkins, Johns Hopkins University School of Medicine, Baltimore, MD 21287 USA

**Keywords:** Skin models, Non-coding RNAs

## Abstract

How developmental programs reactivate in regeneration is a fundamental question in biology. We addressed this question through the study of Wound Induced Hair follicle Neogenesis (WIHN), an adult organogenesis model where stem cells regenerate de novo hair follicles following deep wounding. The exact mechanism is uncertain. Here we show that self-noncoding dsRNA activates the anti-viral receptor toll like receptor 3 (TLR3) to induce intrinsic retinoic acid (RA) synthesis in a pattern that predicts new hair follicle formation after wounding in mice. Additionally, in humans, rejuvenation lasers induce gene expression signatures for dsRNA and RA, with measurable increases in intrinsic RA synthesis. These results demonstrate a potent stimulus for RA synthesis by non-coding dsRNA, relevant to their broad functions in development and immunity.

## Abstract (web summary)

During wound induced hair follicle neogenesis (WIHN), stem cells regenerate hair follicles but how this arises is unclear. Here, the authors show that self-noncoding dsRNA activates the antiviral receptor TLR3 to induce intrinsic retinoic acid, which stimulates WIHN in mice, and in isolated human keratinocyte cells.

## Introduction

Tissue morphogenesis occurs with high fidelity during development, but reactivation of morphogenesis to promote regeneration after injury in adults is rare but a major goal. Inducing regeneration in adults could counter post-injury fibrosis that contributes largely to human morbidity. Large wounds in mice and rabbits result in de novo hair follicle morphogenesis^1,2^. In regeneration, damaged tissues employ endogenous signaling to mobilize the proliferation and differentiation of resident stem cells for repair. Here, we use the model of wound-induced hair neogenesis (WIHN) to identify mechanisms of reactivating developmental pathways in adult regeneration. In WIHN, after full-thickness cutaneous wounds to the depth of skeletal muscle, epidermal stem cells drive not just re-epithelialization, but also hair follicle morphogenesis as during embryogenesis^3,4^. While important advancements have been made on the role of Wnt^1^/Shh^5^ signaling, γ-δ T cells^6^, and myofibroblasts^7^, the mechanisms by which epidermal stem cells induce skin regeneration are still unclear^3^. Recent evidence has shown that antiviral pathways are hallmarks of diverse stem cells^8^. Additionally, we reported that damaged skin activates Toll Like Receptor 3 (TLR3) signaling to induce hair follicle regeneration^9^. Together, these findings suggest that in addition to other pathways, TLR3 signaling as a damage sensor might connect stem cell activity to regeneration.

In separate studies of developmental biology, Retinoic Acid (RA) is known to control appendage development and regeneration^10^, for example in the patterning of the blastema that coordinates salamander limb regrowth after amputation^11^. Underscoring a similarity between limb appendage and hair follicle (termed a skin appendage) development, abnormal RA signaling and accumulation also cause progressive hair loss^12^ and inappropriate hair follicle morphogenesis^13^. Separately, among other uses, RA is commonly used clinically for facial esthetic rejuvenation^14^, as are also a variety of other skin damaging treatments such as dermabrasion or laser resurfacing. These data indicate that RA biologically acts as an essential morphogen in development and regeneration, but, interestingly, its clinical use overlaps that with damage-inducing therapies. This suggests the hypothesis that tissue damage responses—such as from TLR3—and RA might act in overlapping contexts.

In support of a connection between TLR3 and RA pathways is an overlap in known signaling proteins. For example, Signal transducer and activator of transcription 3 (STAT3) and nuclear factor kappa-light-chain-enhancer of activated B cells (NF-kB) stimulate aldehyde dehydrogenase1a3 (ALDH1A3) expression^15^, which convert retinaldehyde to RA. Given that STAT3 and NF-kB are essential downstream targets in TLR3 signaling, this implies that TLR3 activation may stimulate RA synthesis. Therefore, we hypothesize that damage might induce the release of double stranded RNA (dsRNA) that activates TLR3 and its downstream pathways to induce RA synthesis and signaling to promote regeneration in its capacity as a known morphogen. Our objective was to test whether this occurs during WIHN. Here, we demonstrate that indeed TLR3 is necessary and sufficient for RA synthesis, and that RA signaling is necessary for WIHN. We show evidence in both humans and mice that a dsRNA-RA axis is a conserved pathway for promoting rejuvenation or regeneration.

## Results

### Cellular responses to dsRNA or RA highly overlap

**Fig. 1:**
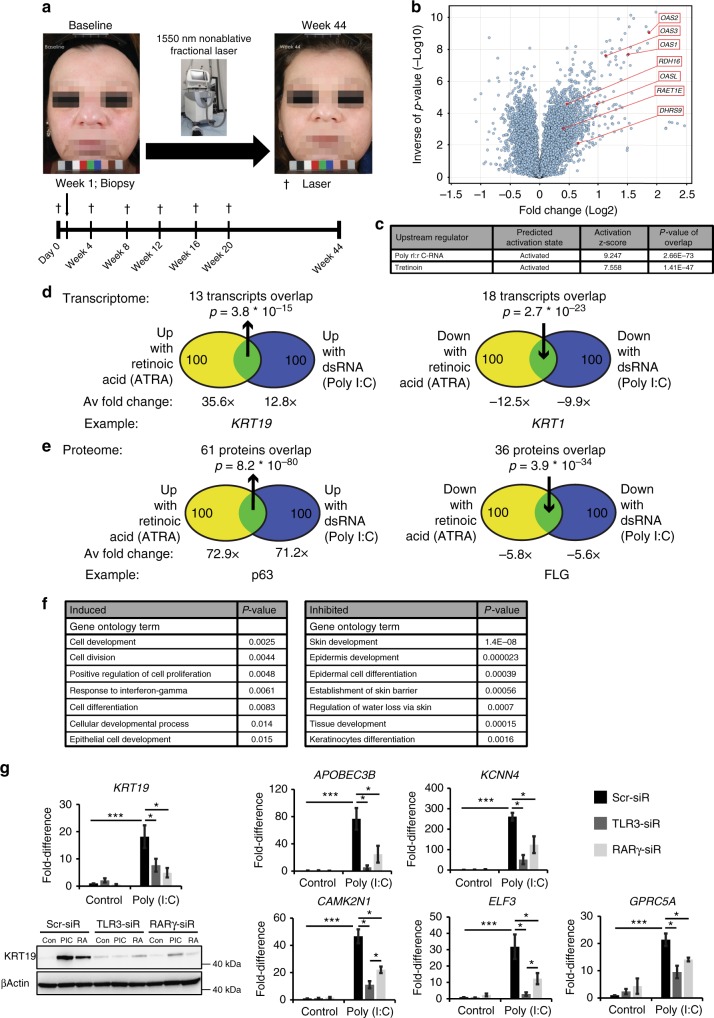
dsRNA and RA cellular response signatures overlap. **a** A 1550 nm nonablative fractional laser treatment improves photoaging. **b, c** After 7 days of laser treatment, among the most induced transcripts are the OAS family (OAS1, 2, 3, and L) and enzymes in RA pathway (RDH16, RAET1E, DHRS9) (**b**); unsupervised upstream regulator analysis confirms strong signatures for dsRNA and Retinoic Acid (RA) induced signaling (**c**). **d, e** Venn diagrams show high overlap in comparisons of top ranked 100 upregulated and downregulated transcripts (**d**; from 49,395) or proteins (**e**; from 3814) between RA- and dsRNA (Poly (I:C); 30 µg per ml)-treated foreskin keratinocytes. **f** Gene ontology analysis of overlap proteins in **e**. **g** Keratinocyte genes upregulated with dsRNA depend on the dsRNA receptor TLR3, but also RARG. Depicted are mRNAs, and western protein analysis of KRT19. (*n* = 3–4 independent experiments, ***P < 0.001, *P < 0.05, one-way ANOVA). Data are means ± SEM

To identify pathways associated with human skin rejuvenation, we treated 17 subjects with a conventional laser treatment known to improve skin photoaging (Fig. 1a) and then biopsied the treated skin to determine gene expression changes 1 week after the first treatment. We noted parallel increases in signatures for the TLR3 ligand dsRNA and RA (Fig. 1a–c), though other immune signatures were upregulated as well (IFNG, TNF, IRF7, IRFNA2). RA is a potent morphogen that is important in development, regulates stem cell function^16^, and itself is a therapy for photoaging, however the mechanism by which its endogenous synthesis is regulated is not fully understood. Therefore, we hypothesized that dsRNA-mediated signaling induces RA synthesis to regulate regeneration, thereby identifying an unexplored functional link between these two pathways in stem cell function, and more broadly in their diverse physiological roles.

We first compared the transcriptomes and proteomes of normal human keratinocytes after treatment with dsRNA (Polyinosinic-polycytidylic acid; Poly (I:C)) or RA in vitro. We ranked the top 100 upregulated and downregulated transcripts (Fig. 1d) and proteins (Fig. 1e) according to their fold change after stimulation and defined the overlapping genes between these two different stimuli. Surprisingly, we found a dramatic overlap. For mRNA transcript expression, Poly (I:C) and RA induced and inhibited 13% and 18% identical gene transcripts, respectively (Fig. 1d). We noted that many of these gene transcripts were involved in keratinocyte differentiation; KERATIN19 (KRT19) is a marker of basal keratinocyte stem cells residing in the hair follicle bulge compartment. Conversely, KERATIN1 (KRT1) is a marker of committed differentiated cells of the suprabasal layer and was inhibited by both stimuli. The overlap was even greater for protein expression, with an overlap of induced and inhibited 61% and 36% respectively (Fig. 1e). Among proteins, we found a similar pattern where genes like tumor protein p63 (TP63/p63) were induced; it is among the earliest transcription factors to define an epidermal fate during development. Similarly, FILAGGRIN (FLG)—a marker of highly differentiated keratinocytes—was inhibited by both stimuli. Unsupervised gene ontology analyses confirmed these findings and demonstrated a substantial enrichment of gene transcripts involved in epidermal development in both induced and inhibited transcripts, with a particular inhibition of keratinocyte differentiation by both stimuli (Fig. 1f). Finally, to begin to test for a functional link between TLR3 and RA signaling, we investigated if transcripts induced by Poly (I:C) require not only its receptor TLR3, but also the RA receptor gamma (RARγ) for induction. We verified this is the case for six transcripts including *KRT19* (Fig. 1g). These findings suggest that dsRNA and RA stimulate highly overlapping cellular responses.

### dsRNA and TLR3 signaling induces RA accumulation

**Fig. 2:**
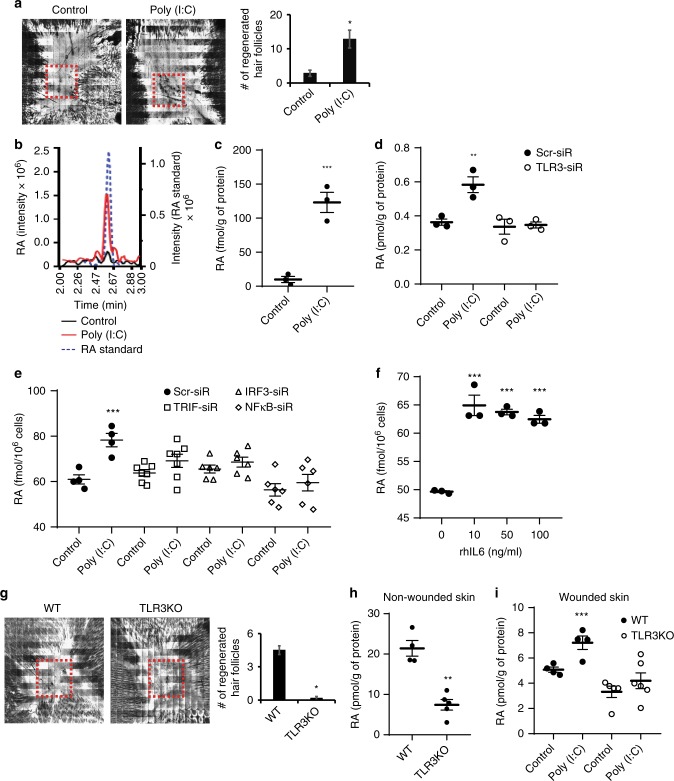
dsRNA and TLR3 are sufficient and necessary to stimulate RA accumulation. **a** A single injection of Poly (I:C) (50 µl; 5 µg per mouse) induces wound-induced hair neogenesis (WIHN) as seen by Confocal scanning laser microscopy (CLSM) images and quantified averages. Red dashed lines indicate area of WIHN (*n* = 5 independent animals, **P < 0.01, unpaired Student *t*-test). **b, c** In LC-MS/MS analysis, Poly (I:C) (0.5 µg per ml) increases RA levels in cell pellet lysates of human scalp keratinocytes) compared to an RA standard (b), and is substantial across replicates (c, *n* = 3 independent experiments, ***P < 0.001, paired Student *t*-test). **d** Poly (I:C) (0.5 µg per ml)-mediated RA synthesis is dependent on TLR3 (*n* = 3 independent experiments, **P < 0.01, one-way ANOVA). **e** Poly (I:C) (0.5 µg per ml) induced RA accumulation requires TLR3 receptor downstream signaling (*n* = 4–7 independent experiments, ***P < 0.001, one-way ANOVA). **f** Recombinant human IL6 induces RA accumulation in human scalp keratinocytes (*n* = 3 independent experiments, ***P < 0.001, one-way ANOVA). **g** Lack of WIHN in *Tlr3*^−/−^ mice. Red dashed lines indicate area of WIHN (*n* = 6, ***P < 0.001, unpaired Student *t*-test). **h, i** *Tlr*3^−/−^ mice have less RA in unwounded (h) (*n* = 4, **P < 0.01, unpaired Student *t*-test) or wounded skin (i) (*n* = 4 independent animals, ***P < 0.001, one-way ANOVA), and are resistant to Poly (I:C) (50 µl; 5 µg per mouse) induced RA accumulation. Data are means ± SEM

Given the overlapping transcripts and proteins regulated by both Poly (I:C) and RA, and the ability for Poly (I:C) to induce WIHN (Fig. 2a), we hypothesized that dsRNA might induce RA synthesis to explain the shared response. To test this, we treated human keratinocytes with Poly (I:C) and collected cell lysates and culture media to measure RA levels. Poly (I:C) markedly increased RA abundance (Fig. 2b, c). To determine the impact of Poly (I:C) on retinoid homeostasis, we also measured retinol (ROL; the substrate for the first step of RA biosynthesis) and retinyl ester (RE; the storage form of vitamin A) but their quantities were not modified as it was for RA (Supplementary Fig. 1b, c). In the case of RA, Poly (I:C)-induced accumulation is dependent on TLR3 (Fig. 2d), analogously to the requirement for TLR3 in WIHN in vivo (Fig. 2g). Consistent with this finding, we also found that TLR3 downstream factors including TIR-domain-containing adapter-inducing interferon β (TRIF/TICAM1), interferon regulatory factor 3 (IRF3), and NF-kB were required and Interleukin 6 (IL-6) sufficient to substantially stimulate RA accumulation (Fig. 2e, f). These results suggest that dsRNA through TLR3 signaling induces intrinsic RA synthesis at its most distal step to promote the conversion from retinal to RA. These observations raised the questions of whether this occurs in vivo.

To study the in vivo interplay between dsRNA and RA, we focused on WIHN given the quantitative ability to count de novo follicles in a defined wound size as an index of regeneration. TLR3 is an important dsRNA receptor in wounding and WIHN^9,17,18^. After wounding, Poly (I:C) stimulates new hair follicle formation (WIHN) (Fig. 2a), whereas *Tlr3*^−/−^ mice show limited regeneration capacity compared to wild-type (WT) mice (Fig. 2g). Given that Poly (I:C) induces intrinsic RA synthesis, we speculated that *Tlr3*^−/−^ mice might have lower RA abundance. We first measured endogenous RA levels using non-wounded normal skin. As expected, WT mice had more RA at baseline than *Tlr3−*^/−^ mice (Fig. 2h), while other metabolites were not substantially different (Supplementary Fig. 1h–i). Moreover, the amount of RA, but not other metabolites (Supplementary Fig. 1j–k), was substantially increased during wound healing by Poly (I:C) treatment in WT mice, but much less so in *Tlr3*^−/−^ mice (Fig. 2i). As seen in vitro, these findings demonstrate in both unwounded basal tissue and in response to exogenous dsRNA, TLR3 is required for RA synthesis in vivo.

### RA induces hair follicle regeneration in Tlr3 deleted mice

**Fig. 3:**
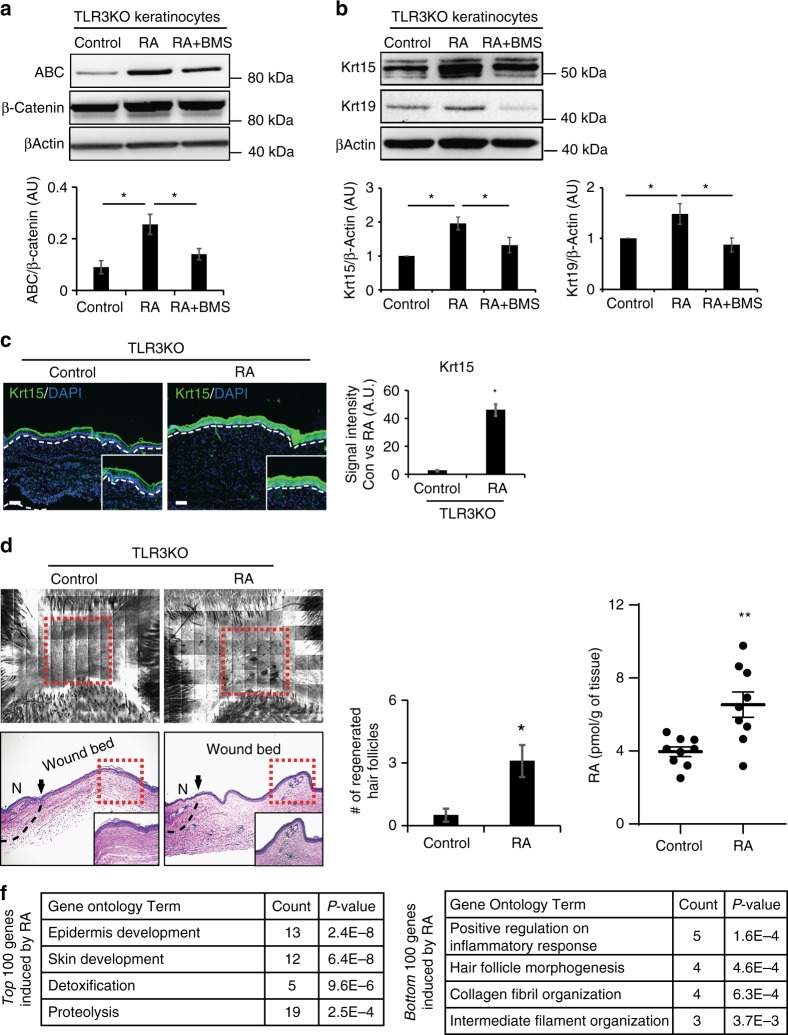
RA induces keratinocyte stem cell markers and promotes WIHN in *Tlr*3^−/−^ mice. **a** RA induces active β-catenin (ABC) expression in keratinocytes of *Tlr*3^−/−^ mice, while the pan-RAR antagonist BMS493 inhibits induction. Representative western blot (top) and quantitation (bottom), normalized to total β-catenin. (*n* = 3 independent experiments, *P < 0.05, paired Student *t*-test). **b** RA induces Krt15 and Krt19 expression in keratinocytes of *Tlr*3^−/−^ mice, while BMS493 substantially inhibits expression. Representative western blot (top) and quantitation of Krt15 (bottom left) and Krt19 (bottom right), normalized to β-Actin. (*n* = 3 independent experiments, *P < 0.05, paired Student *t*-test). **c** RA induces prominent Krt15 (green) in *Tlr*3^−/−^ wounds as detected by immunostaining (left) and quantitated (right; *n* = 3 independent animals, ***P < 0.001, paired Student *t*-test). White dashed lines indicate basement membrane. Nuclei stained with DAPI (blue). White scale bar = 100 µm. **d** RA induces WIHN in *Tlr*3^−/−^ mice as detected by CLSM (top), H&E staining (middle) and quantification (right; *n* = 6–11 independent animals, *P < 0.05, unpaired Student *t*-test). Black arrows and dashed lines signify the boundary between unwounded and wounded area. Red dashed lines indicate area of WIHN. Black scale bar = 100 µm. N; normal unwounded skin. **e** Exogenous RA treatment increases RA levels in *Tlr*3^−/−^ mice (*n* = 9 independent animals, **P < 0.01, paired Student *t*-test). **f** Gene Ontology analysis of the top and bottom 100 transcripts after RA treatment to *Tlr*3^−/−^ mice. Data are means ± SEM

Given that *Tlr3*^−/−^ mice have lower levels of endogenous RA and less WIHN compared to WT mice, our next question was whether the impaired regeneration of *Tlr3*^−/−^ mice could be improved with exogenous RA treatment. We first isolated keratinocytes or whole skin from *Tlr3*^−/−^ mice and noted that RA increased the levels of Krt19, Krt15, and activated β-catenin protein, known to be necessary and sufficient for WIHN^1,19^ (Fig. 3a–c). Concomitantly, exogenous RA modestly increased hair follicle regeneration in *Tlr3*^−/−^ mice (Fig. 3d), with measurable increases in tissue RA levels prior to morphogenesis (Fig. 3e). While the increase was significant (unpaired *t*-test), the background strain of these mice prohibits high levels of WIHN^9^, and known induction of RA degradation pathways by RA might explain the modest increase. However, in microarray analysis of *Tlr3*^−/−^ mice treated with RA, we identified a substantial enrichment of gene ontology categories pertaining to development and morphogenesis (Fig. 3f), as seen during regeneration^9^. Collectively, these results demonstrate that dsRNA and TLR3 are responsible in vivo for RA production and hair follicle regeneration.

Our next aim was to define the mechanism of how dsRNA and TLR3 increase RA synthesis. To this end, we noted highly synergistic induction between Poly (I:C) and RA of transcription for key players thought to stimulate WIHN. Expression of KRT15, TLR3, Wingless and the name Int-1-7b (WNT7b), and Ectodysplasin A receptor (EDAR) were potently upregulated by a combination of RA and Poly (I:C) in cultured human keratinocytes and human skin explants (Supplementary Fig. 3a–c). Given the potency of combined RA and Poly (I:C), we hypothesized that an RA synthesis positive feedback loop enhances intrinsic RA synthesis and function. We therefore tested three enzymes known to convert retinal to RA (Aldehyde dehydrogenase 1 family, member A1-A3; ALDH1A1-A3) for synergistic response to RA and Poly (I:C) in human keratinocytes.

### dsRNA induces RA synthesizing enzymes for RA accumulation

**Fig. 4:**
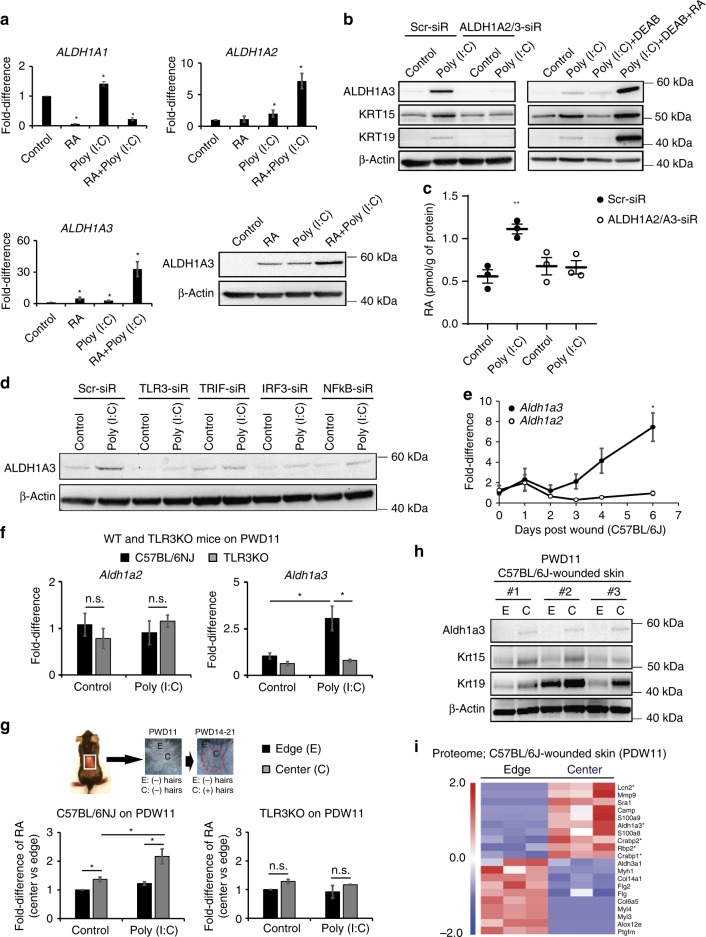
dsRNA induces ALDH1A2/A3 for RA synthesis and accumulation. **a** RA and Poly (I:C) (0.1 µg per ml) synergistically induce ALDH1A2 and A3 transcription, but not A1 as detected by qRT-PCR in human scalp keratinocytes. (*n* = 3 independent experiments, *P < 0.05, paired Student *t*-test). Western blot staining shows ALDH1A3 induced by RA and Poly (I:C) (*n* = 2). **b** Genetic (left; siRNA) or chemical (right; DEAB compound) inhibition of ALDH1A2/A3 decrease the ability of Poly (I:C) (0.1 µg per ml) to induce KRT15 and KRT19 in human scalp keratinocytes as detected by western blotting (*n* = 2 independent experiments). **c** Genetic inhibition of ALDH1A2/A3 in human keratinocytes reduces RA synthesis in presence of Poly (I:C) (0.5 µg per ml) (*n* = 3 independent experiments, *P < 0.05, one-way ANOVA). **d** TLR3 and its downstream mediators are required for stimulation of ALDH1A3 in human scalp keratinocytes (*n* = 3 independent experiments). **e** Expression of *Aldh1a2* and *Aldh1a3* in WT mice during wound healing (*n* = 3–4 independent animals, *P < 0.05, unpaired Student *t*-test). **f** *Aldh1a3* but not *Aldh1a2* is induced in wild type mice by Poly (I:C) (50 µl; 5 µg per mouse) in early wounds; neither is induced in the *Tlr*3^−/−^ mice as detected by qRT-PCR (*n* = 3 independent animals, *P < 0.05, paired Student *t*-test). **g** Schematic of sampling procedure (top). Before morphogenesis, the center (C; high future regeneration) of the wound has more RA than the edge (E; lower future regeneration) and accumulates more RA in response to Poly (I:C) (50 µl; 5 µg per mouse) in wild type (left) but not *Tlr*3^−/−^ mice (right) as measured by LC-MS/MS (right; *n* = 4 independent animals, *P < 0.05, paired Student *t*-test). White solid box indicates wounded area and red dashed lines signify center region of wound-healed skin. **h** Before morphogenesis, the center of the wound expresses more Aldh1a3, Krt15, and Krt19 than the edge in WT mice (*n* = 3 independent animals). **i** Increased RA pathway proteins in proteomic analysis of the C vs. E of the wounded skin prior to morphogenesis (*n* = 3 independent animals). * Indicates factors for RA pathway. Data are means ± SEM

*ALDH1A1* showed the least robust response and no synergistic activation to RA and Poly (I:C). However, *ALDH1A3* and *ALDH1A2* to a lesser extent were induced by RA and Poly (I:C) (Fig. 4a). ALDH1A3 protein was robustly increased by either RA or Poly (I:C), but particularly with both (Fig. 4a). To define the functional importance of ALDH1A2 and A3, we inhibited ALDH1A2/A3 in human keratinocytes with either siRNA or broad ALDH chemical inhibition (N,N-diethylaminobenzaldehyde; DEAB). Normally following Poly (I:C) stimulation, ALDH1A3 and the hair follicle stem cell markers KRT15 and KRT19 protein expression are upregulated, but this induction was disrupted with both methods of ALDH1A2/A3 inhibition (Fig. 4b). Moreover, Poly (I:C)-induced RA synthesis is dependent on ALDH1A2/A3 (Fig. 4c). Supporting this, TLR3 downstream genes are required for Poly (I:C) induction of ALDH1A3 in human keratinocytes (Fig. 4d). In wild type mice, ALDH1A3 is induced during late stage wounding, while ALDH1A2 is not (Fig. 4e). Indeed, in WT but not *Tlr3*^−/−^ mice, Poly (I:C) induced *Aldh1a3* transcription during wound healing, while *Aldh1a2* did not show a similar pattern (Fig. 4e, f). Taken together, these results demonstrate the functional importance of ALDH1A2/A3 in TLR3/dsRNA-induced stem cell marker induction and RA synthesis.

During WIHN, neogenic hair follicles form in the center of the wound and less so at the periphery. This patterning is reminiscent of developmental processes; RA defines the anterior/posterior axis by forming morphogen gradients during embryo development^10^. We therefore measured RA abundance in center versus edge skin areas after wound closure, but prior to the initiation of neogenic follicles (Post Wound Day 11; PWD11). Interestingly, RA levels were higher in the center and lower at the periphery (Fig. 4g), so that higher RA levels were detected in areas that later harbored more WIHN; conversely, lower RA levels were detected in areas that later harbored less WIHN. Consistent with this, at early pre-regeneration time-points, the center of wound expressed more Aldh1a3, Krt15, and Krt19 protein than the periphery (Fig. 4h). Proteomics confirmed an abundance of RA-mediated signaling proteins in the center area of healed skin prior to regeneration (Fig. 4i). Therefore, dsRNA increased the abundance of RA, particularly in this center region, to establish an RA concentration differential for regeneration, suggesting that RA might directly enhance WIHN.

### Rarα is necessary for baseline and dsRNA-augmented WIHN

**Fig. 5:**
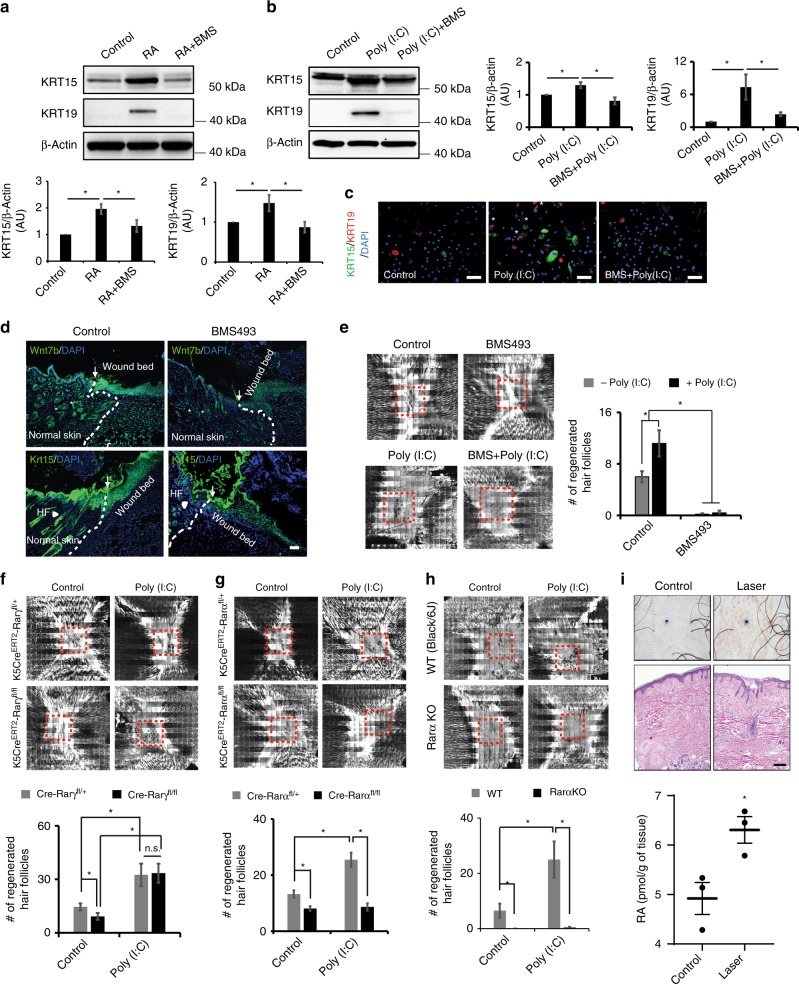
Rarα is required for baseline and dsRNA augmented WIHN. **a**, **b** Chemical inhibition of RARs (BMS493) decreases RA (left)- and Poly (I:C) (0.1 µg per ml) (right)-induced KRT15 and KRT19 expression in human scalp keratinocytes as detected by western blot with quantitation (*n* = 3 independent experiments, *P < 0.05, paired Student *t*-test). **c** Immunostaining of KRT15 (green), KRT19 (red) and nuclei (DAPI; blue). * Represents co-stained cells of KRT15 and KRT19. White scale bar = 100 µm. **d** BMS493 inhibited Krt15 and Wnt7b expression in healing wounds as detected by immunostaining. White dashed lines and arrows separate normal and wounded areas. White arrow heads indicate hair follicles (HF) in normal skin. White scale bar = 100 µm. **e**, BMS493 treatment inhibited WIHN, in untreated or Poly (I:C) (50 µl; 5 µg per mouse)-treated WT mice. Red dashed lines indicate area of WIHN. (*n* = 5 independent animals, *P < 0.05, paired Student *t*-test). **f**–**h** Conditional epithelial specific deletion of Rara (**g**; *n* = 4-9 independent animals, *P < 0.05, paired Student *t*-test) and global deletion of Rara (**h**; *n* = 4–11 independent animals, *P < 0.05, paired Student *t*-test) both inhibit baseline and Poly (I:C) (50 µl; 5 µg per mouse) augmented WIHN, while epithelial deletion of Rarg has minimal effects (**f**). Red dashed lines indicate area of WIHN. **i** Rejuvenating fractional ablative CO_2_ laser induces RA synthesis in human scalp (*n* = 3 independent human samples, *P < 0.05, paired Student *t*-test; bottom). Clinical photos (top) and H&E histology (middle) of human subjects before and after CO_2_ laser treatment. Black scale bar = 1 mm. Data are means ± SEM

Having defined the functional importance of dsRNA, TLR3, ALDH1A2/3 and RA, we next explored the mechanism by which RA controls regeneration. RA binds its receptors (RAR-alpha, beta, and gamma; RARα, RARβ, RARγ) to form heterodimers with retinoid X receptors (RXRs) and initiate gene transcription^20,21^. To investigate whether Poly (I:C)-induced RA signaling is required for hair follicle regeneration, we first inhibited RA signaling using a pharmacological pan-RAR antagonist (BMS493) in the presence of Poly (I:C). BMS493 substantially inhibited RA and Poly (I:C)-induced KRT15 and KRT19 expression in human keratinocytes (Fig. 5a–c). We next investigated RAR inhibition in vivo. BMS493 treatment in WT mice decreased Wnt7b and Krt15 protein expression in wound beds prior to regeneration (Fig. 5d). Consistent with this, BMS493 treatment almost completely ablated all WIHN (Fig. 5e), with or without Poly (I:C). These results suggest that dsRNA-induced RA signaling after wounding is important to promote hair follicle neogenesis.

Given the potential off-target effects of pharmacologic inhibition, we used genetic targeting to determine which specific RAR is important for regeneration. Since dsRNA/TLR3 signaling induces RA synthesis in human keratinocytes that express both RARα and RARγ, but not RARβ^22,23^, we measured WIHN in keratinocyte-targeted Rarα and Rarγ null mice (Krt5-CreERT2-Rarα^fl/fl^ and Krt5-CreERT2-Rarγ^fl/fl^). Keratinocyte-specific Rarγ deficient mice displayed mostly intact hair follicle regeneration (Fig. 5f) whereas the homozygous Rarα null mice (Krt5-CreERT2-Rarα^fl/fl^) receiving vehicle or Poly (I:C) had a marked inhibition of hair follicle formation, compared to heterozygous mice (Fig. 5g). The effect of Rarα deletion on hair follicle regeneration was even more pronounced in global Rarα null mice (Fig. 5h), possibly due to deletion in other contributing cell types, to more efficient gene deletion in keratinocytes, or both. Underscoring the possibility of cooperativity, combined RARα/γ deletion in keratinocytes also exhibited less WIHN (Supplementary Fig. 4). These results demonstrate the importance of retinoic acid receptors in responding to RA and enhancing regeneration.

### Laser treatment induces RA accumulation in human subjects

Finally, to assess the human relevance of these findings, we treated human subjects with a laser normally used for facial rejuvenation, and again discovered elevations in RA synthesis (Fig. 5i). These results demonstrate the importance of RA in the regulation of normal and dsRNA induced regeneration. Moreover, these data indicate that these conserved pathways despite highly distinct contexts are upregulated in common cosmetic procedures and highlight how dsRNA/damage induction of RA synthesis is central to rejuvenation therapies.

## Discussion

Antiviral inflammatory pathways are a conserved feature of multiple types of stem cells, but their nonimmune function is unclear^8^. Also, RA has been demonstrated to be important for hematopoietic stem cell dormancy, limb development and even limb regeneration^10,11,24^. However, how these seemingly disparate features might be connected has not been clear. Based on TLR3 enhancement of healing after injury^9,25,26^, we discovered that dsRNA/TLR3 stimulates a RA morphogen-like pattern concentration differential to promote stem cell function in hair follicle neogenesis. These results suggest a broader and previously unrecognized connection of noncoding dsRNA sensing and RA signaling during development, regeneration, and likely normal functions.

The mechanistic link between damage induced dsRNA and RA synthesis that we demonstrate matches the clinical commonality of damage-based treatments and RA used in dermatology for skin rejuvenation. A wide variety of treatments such as dermabrasion, microneedling, peels, and lasers reverse fine wrinkling and pigmentary changes associated with photoaging. RA succeeds in this regard. Our current study implies that all of these therapies might work through the same final common pathway of activating RARs. Indeed, clinicians also have made the observation that RA pretreatment enhances outcomes in damage based treatments^27^. These clinical observations dovetail with our findings of synergistic transcriptional responses in combined RA and dsRNA treated cells (Fig. 4 and Supplementary Fig. 3). Therefore, our present findings provide a mechanistic explanation for these previous clinical observations and suggest future studies to examine whether controlled damage and RA can enhance tissue remodeling in organs other than skin.

RA has broad uses in medicine, for example in the treatment of acute promyelocytic leukemia. However, in all contexts, treatment can result in poor efficacy and significant side effects. This might partly be due to the artificial nature of exogenous RA delivery in these clinical settings; inducing endogenous synthesis might be far more effective, for example because of the simultaneous induction of chaperones. In support of this, we find that in conditions where ALDH1A3 is maximally induced, such as with RA plus Poly (I:C) (Fig. 4a), stem cell gene induction (Supplementary Fig. 3) is also maximally induced—up to ~250 fold more than with exogenous RA treatment alone. This implies that methods of inducing nuclear steroid hormone ligand production will be more efficacious than simple ligand addition in their respective clinical indications.

Our results raise other interesting questions. One is the potential role of non-TLR3 dsRNA sensors. For example, retinoic acid-inducible gene I (RIG-I/DDX58) and melanoma differentiation associated gene-5 (MDA5/IFIH1) recognize short and long dsRNAs, respectively^28^. We find that mice deficient in RIG-I or MDA5 are still capable of WIHN, though the background strain of RIG-I is unusually low (Supplementary Fig. 7a, b). Moreover, human keratinocytes deficient in these genes still induce RA synthesis after PIC treatment (Supplementary Fig. 7c–e), but this is not the case in TLR3 deficient cells (Fig. 2d, h, i). Another question is related to the effect of supraphysiologic doses of RA in WIHN. Although RA is sufficient to increase WIHN in *Tlr3*^−/−^ mice (Fig. 3d), and RA is required for WIHN in WT animals (Fig. 5), supraphysiologic doses of RA do not modify WIHN (Supplementary Fig. 6). This occurs in other contexts of RA biology, and might be due to lack of chaperones or induction of degradation pathways that tightly regulate this potent biologic pathway. Biologic pathway differences might also relate to the differences we see in how RA abundance changes in different types of wounds; after human laser wounding, RA increases while the absolute level of RA appears lower in wounded mouse skin compared to unwounded skin. Perhaps the most likely explanation appears to be that hair follicles are rich in RA abundance, and their numbers are unchanged after human laser wounding, but dramatically decrease after full thickness skin excision in our mouse model. Future work should investigate the contribution of adnexal structures to RA abundance, endogenous RA pathway regulation, the likely nonlinear pathways regulating regeneration, and other non-TLR3 pathways in WIHN.

Altogether our results demonstrate that dsRNA/TLR3 stimulates preferential RA accumulation in a pattern that correlates with future hair follicle neogenesis, reminiscent of morphogen signaling. Although there are differences between human facial rejuvenation and mouse WIHN, here we demonstrate how noncoding dsRNA activates TLR3 to trigger RA synthesis and signaling to promote regeneration. Our work implies that therapeutic combination of TLR3 agonists with RA might synergistically enhance regeneration in humans.

## Methods

### Human samples

Hopkins IRBs (NA_0033375, NA_00075350, IRB00028768) and Helsinki principles were followed. Skin was processed as described^29,30^. Human biopsies were donated under written informed consent and Hopkins IRB approved protocol (NA_00033375) following Declaration of Helsinki principles. Discarded skin from Mohs surgery or foreskin excisions was used for ex vivo explant experiments and keratinocytes isolation. Skin tissues were prepared for generating paraffin embedded or frozen tissue sections.

### Human photoaging study

Under Hopkins IRB (IRB00028768) with all relevant ethical regulations for work with human participants, 17 Caucasian women Fitzpatrick types I–III with average age of 55 and moderate to severe baseline photoaging were enrolled after informed consent including permission to publish skin photography in Fig. 1. Laser treatments and biopsy schedule were as listed, with treatments occurring both to the face and arm, and arm biopsies used for gene expression data listed. The authors affirm that human research participants provided informed consent for publication of the images in Fig. 1a.

### Human RA measurement study

Under Hopkins IRB (NA_00075350), 3 human subjects with Central Centrifugal Cicatricial Alopecia with large areas of scarring hair loss were enrolled. Two analogous areas of alopecia were identified. Both were injected with 1% lidocaine with epinephrine, tattooed and photographed. The first was biopsied using a 4 mm punch tool and used as the pretreatment sample. The second was treated with a single pass of the Lutronic eCO_2_ laser (120 hand piece; 30 W; 240 mJ; 100 density, 12 × 12 mm, Static mode). After 1 week, the subject returned for a repeat photography and biopsy as above. The samples were processed for mass spectrometry as described below. Histology is from before and 30 days after laser treatment.

### Adult keratinocytes isolation and cultures

For human keratinocytes, 3-mm skin biopsies were taken from areas of the occipital scalp through above IRB protocols using enzyme digestion method as described in the literature^30,31^. For mouse keratinocytes, tails of adult mice were used and followed by the same protocol of human keratinocytes. Briefly, the skin fragments were incubated in 0.4% dispase II solution overnight at 4°C, allowing separation of epidermis from dermis. Then epidermis was incubated with 0.25% trypsin-EDTA (Lonza, Walkersville, MD) to isolate keratinocytes for 5 min at 37 °C. Adult keratinocytes were maintained in KGM including defined growth factors (KGM-GOLD Bullet kit, #192060) (Lonza) and supplemented with 10 µM of ROCK inhibitor (Y-27632) (Cayman Chemical, #10005583) until use^32^. For all in vitro experiments, primary keratinocytes were passaged at least once to eliminate contaminating other cells isolated from epidermis and used at passage 2–4 for human and passage 1–2 for mouse keratinocytes.

### Mouse strains

All mouse experiments were approved by JHU IACUC (MO17M298). Mouse strain, genotype and treatment regiments can be found in Supplementary Table 3. C57BL/6NJ (JAX stock #005304) and C57BL/6J (JAX stock #00664) were used as the wild type strains for all in vivo experiments. Tlr3 (B6N.129S1-Tlr3^tm1Flv/^J; stock #009675), Mda5 null (B6.Cg-Ifih1^tm1.1Cln^/J; stock #015812), and Rarα null mice (Rara^tm1Rev^/HsvJ; stock #023845) were obtained from the Jackson Laboratory. RIG-I null and wild type mice in 129Sv/C57BL/6 background were provided by the Adolfo Garcia-Sastre laboratory (New York, NY). Krt5-CreERT2, Rarα-flox and Rarγ-flox mice were kindly provided by Pierre Chambon and Krt5-CreERT2 mice were crossed with mice including Cre-dependent floxed Rarα^fl/fl^ and Rarγ^fl/fl^. DNA was extracted from tail tips of all Cre-LoxP transgenic mice and genotyped by PCR using the Red-Xtract Kit (Millipore Sigma, XNAT-100) and appropriate primer sets (Supplementary table 1). For Cre-ERT2 transgenic mice, tamoxifen (Millipore Sigma, T5648) was dissolved in corn oil at 10 mg per ml and 75 mg of tamoxifen per kg of body weight was injected intraperitoneal (i.p.) every day for 9 days from day 15–24^33^. Mice were maintained by animal facilities at Hopkins and all experiments were performed by animal protocols (#MO17M298) approved by the Johns Hopkins University Animal Care and use Committee.

### Wound-induced hair neogenesis

All experiments were followed with our (WIHN protocol^1,9^. Briefly, hair was shaved and a 1.2 cm^2^ full-thickness skin on the backs of 21-day old (telogen) male and female mice was removed by sterile scissors. A single treatment of RA (30 µl) (0.1 µg per mouse) (Millipore Sigma, R2625) and BMS493 (30 µl) (0.1 µg per mouse) (Cayman Chemical, #17418) was applied topically onto open wound on WD1 of *Tlr*3^−/−^ and WT mice, respectively. Poly (I:C) (50 µl) (5 µg per mouse) (Invivogen, tlrl-pic) was injected under the healed scab on WD3 of WT mice as described before^9,34^. On WD20-24, numbers of regenerated hair follicles were visualized and quantified in the re-epithelialized skin by noninvasive confocal scanning laser microscopy (CSLM) (Caliber I.D., VIVASCOPE 1500) as published^1,9^. As a vehicle (Control), ethanol (EtOH), dimethylsulfoxide (DMSO), or PBS was treated for RA, BMS493, and Poly (I:C), respectively.

### Microarray analysis

RNA isolated from human foreskin keratinocytes with or without Poly (I:C) (30 µg per ml) (GSE92646) and mouse wound tissues from wild type (C57BL/6NJ) and *Tlr3*^−/−^ with BMS493 and RA were submitted to the JHMI Deep Sequencing & Microarray core for Affymetrix^®^ Human Exon 1.0ST and mouse microarray chip according to manufacturer’s protocols. Raw gene expression signals in the form of Affymetrix CEL files were extracted and normalized with Partek^®^ Genomics Suite™ software using the Robust Multichip Analysis (RMA) algorithm. Genes were ranked according to fold change of the intervention, and referred to as top or bottom induced if they are at the highest fold change (top) or lowest fold change (bottom). The significance of gene expression was measured by Student’s *t*-test analysis. These datasets generated during the current study are available in the NCBI GEO repository as listed above.

### Small interference RNA transfection

Human ALDH1A2 (Santa Cruz Biotechnology, sc41443), ALDH1A3 (Santa Cruz Biotechnology, sc43611), TLR3 (Dharmacon, M-007745-00), TRIF (Santa Cruz Biotechnology, sc106845), IRF3 (Santa Cruz Biotechnology, sc35710), NF-κB (Santa Cruz Biotechnology, sc29410) and nontargeting control (scramble) siRNAs (Santa Cruz Biotechnology, sc37007 & Dharmacon, D-001210) were commercially provided. Human scalp keratinocytes (3 × 10^5^ cells per well) in 12-well plates were transfected with 20 nM of siRNAs using Lipofectamine^®^ RNAiMAX (Thermo Fisher Scientific, #13778150) following the manufacturer’s directions. Poly (I:C) (0.1 µg per ml) was applied to transfected cells and incubated for 48 h. At the end of transfection period, cells were harvested to isolate protein to perform qRT-PCR, western blot, and RA mass spectrometry. We note that different doses of poly (I:C) were used in separate experiments for technical reasons. For example, after siRNA treatment, cells are more susceptible to poly (I:C) for exact reasons unknown but likely related to increased membrane permeability and associated toxicity given the cellular stress of transfection after transfection for siRNA treatment, so necessitating a lower dose. See figure legends for dose used in each experiment.

### BMS493 treatment

BMS493 pan-RARs antagonist (1 µM) was pre-treated into keratinocytes for an hour and cells were incubated with RA (0.1 µM and 0.5 µM for human and mouse keratinocytes, respectively) or Poly (I:C) (0.1 µg per ml for both human and mouse keratinocytes) for 48 h. At the end of the experiment, cells were harvested for RNA and protein extraction to do further analysis including qRT-PCR and western blot.

### IL6 treatment

Recombinant human interleukin 6 (rhIL6) (R&D system; 206-IL-010) was treated into human scalp keratinocytes with dose-dependent manners (0, 10, 50, and 100 ng per ml) for 2 days. Cells were harvested and lysates were used for RA measurement.

### Tissue preparation from wounded mice

Wild type (C57BL/6J and 6NJ) and *Tlr*3^−/−^ mice were wounded as did in WIHN experiments. On PWD11 when wounds are closed, the center and edge of the wound was separated by scissors. Skin samples were immediately frozen and saved in liquid nitrogen for further analysis.

### Proteomic analysis

For in vivo samples, wild-type and *Tlr3*^*−/−*^ unwounded and wounded skin where wound regions (center and edge area) were prepared for analysis. For in vitro samples, human primary scalp keratinocytes treated with 0.1 µM RA or 0.5 µg per ml Poly (I:C) and control were used. In vivo and in vitro samples were washed in phosphate-buffered saline (PBS) and lysed in 5% sodium deoxycholate. Lysates were further prepared with following steps including washing, reducing, alkylating and trypsinolyzing on filter as explained by Wisniewski et al.^35^ and Erde et al.^36^ For separating and analyzing tryptic peptides, we used a nano ACQUITY UPLC BEH130 C18 column (1.7 μm, 75 μm x 200 mm, Waters) using linear gradient from 3% to 40% acetonitrile with 0.1% formic acid over a 165 min on a Waters nano-ACQUITY UPLC system coupled with Orbitrap Fusion Tribrid mass spectrometer (Thermo Scientific) as described by Williamson et al.^37^ A full scan was acquired at a resolution of 120,000, and fragmentation of precursor ions were selected by higher-energy collisional dissociation with a normalized collision energy at 30% for a maximum 3-s cycle. MS Amanda algorithm was used to search tandem mass spectra against a UniProt human reference proteome database^38^. We set a maximum precursor mass error tolerance of 10 ppm and carbamidomethylation of cysteine and deamidation of asparagine and glutamine as static and dynamic modifications, respectively. For validation of resulting hits, a semi-supervised machine learning algorithm Percolator was used with a maximum false discovery rate of 0.01^39^. To calculate protein abundance ratios, we compared the MS1 peak volumes of peptide ions, which identified by confirming MS2 sequencing as described above. An aligned accurate mass and retention time (AMRT) cluster quantification algorithm Minora (Thermo Fisher Scientific, 2017) was used for the Label-free quantifications. Pathway and gene ontology analysis were conducted with Qiagen Ingenuity, Panther GO and DAVID databases, as reported by Krämer et al.^40^, Mi et al.^41^, and Huang et al.^42^, respectively.

### Determination of retinoid levels

For in vitro samples, human scalp keratinocytes were incubated with rhIL6 or Poly (I:C) (0.5 µg per ml) for 48 h or siRNA-transfected cells were incubated with Poly (I:C) (0.1 µg per ml) for 48 h. For in vivo samples, skin was analyzed from wild-type and *Tlr3*^−*/*−^ unwounded and wounded skin where wound regions (center and edge area) were cut by scalpel. Human skin was analyzed for retinoids with and without CO_2_ laser treatment as described. All samples were frozen at collection and stored at −80 °C until extraction. For in vitro experiments, media and cell pellets were analyzed separately. For in vivo experiments, tissues were homogenized in saline. Extraction of retinoids was performed under yellow lights using a two-step liquid–liquid extraction procedure using 4,4-dimethyl-RA as an internal standard RA and retinyl acetate as an internal standard retinol (ROL) and total retinyl ester (RE)^43–46^. For the extraction of retinoids, 1–3 ml of 25 mM KOH in ethanol was added to tissue lysates (in vivo) or cell culture (in vitro) samples. Then 10 ml hexane (for in vivo tissue sample) or 5 ml hexane (for in vitro cell culture sample) was loaded to the aqueous ethanol phase. The samples were vortexed and centrifuged for 1–3 min at 1000 rpm to separate the aqueous phase from protein pellets. The top hexane phase including ROL and RE (nonpolar retinoids) was discarded. After the remaining aqueous ethanol phase was mixed with 60–180 μl of 4 M HCl solution, polar retinoids (RA) were obtained by extraction with a second 5–10 ml of hexane as described above. Organic hexane phases were evaporated under gaseous nitrogen while heating at 25–30 °C in a water bath (model N-EVAP 112, Organomation Associates, Berlin, MA, USA). All samples were resuspended in 60 μl of analytical grade acetonitrile. Only glass products such as pipetts, syringes, and containers in handling retinoids were used to avoid artificial degradation.

Levels of RA were determined by multiple reaction monitoring cubed (MRM^3^)-based liquid chromatography (LC)-mass spectrometry (MS) which is an LC-MS/MS method utilizing two distinct fragmentation events for enhanced selectivity^43^. RA was measured using a ultrafast liquid chromatography (UFLC) system (Shimadzu Prominence UFLC XR, Shimadzu, Columbia, MD) coupled to an AB Sciex 5500 or 6500 + QTRAP hybrid triple quadrupole mass spectrometer (TQMS) (AB Sciex, Framingham, MA) using atmospheric pressure chemical ionization (APCI) performed in positive ion mode^43^. For the LC separation, the column temperature was regulated at 25 °C, the auto-sampler was maintained at 4 °C, and 10–20 μl was typically injected. All separations were conducted using a reverse phase-amide guard cartridge column (Ascentis Express RP-amide guard column, 50 × 2.1 mm, 2.7 μm, Supelco) coupled to a reverse phase-amide analytical column (Ascentis Express RP-amide column, 100 × 2.1 mm, 2.7 μm, Supelco). Mobile phase A containing 0.1% formic acid in water, and mobile phase B containing 0.1% formic acid in acetonitrile. Endogenously occurring retinoid isomers including all-trans-retinoic acid (atRA), 9-*cis* RA, 13-*cis* RA, and 9,13-di*-cis* RA are resolved using a gradient separation at a flow speed of 0.4 mL per min with the following gradient: 0−1 min, 60% B; 1–7 min, ramp to 95% B; 7–9 min, hold at 95% B; 9–9.5 min, ramp to 10% B; 9.5–10.5 min, hold at 10% B; 10.5–11 min, ramp to 95% B; 11–12.5 min, hold at 95% B; 12.5–13 min, ramp to 60% B; 13–15 min, re-equilibrate at 60% B. The APCI source conditions and MRM^3^ detection parameters were as follows:curtain gas, 15; collision gas, low; nebulizer current, 3; temperature, 325; ion source gas, 55;declustering potential, 56; entrance potential, 12; collision energy, 17; excitation energy, 0.1; excitation time, 25; ion trap fill time, 125. Final MRM^3^ transitions for RA were *m/z* 301.1 → *m/z* 205.1 → *m/z* 159.1 and for 4,4-dimethyl RA were *m/z* 329.2 → *m/z* 151.2 → *m/z* 100.0.

Retinol and RE were quantified via HPLC-UV according to previously published methodology^46,47^. ROL and RE were analyzed by reverse-phase chromatography (Zorbax SB-C18, 4.6 × 100 mm, 3.5 μm) on an Acquity UPLC H-class system (Waters) and were quantified by UV absorbance at 325 nm. Analytes were separated at a flow speed of 1 ml per min with 11% water:89% acetonitrile:0.1% formic acid for 9 min, followed by a linear gradient over 2 min to 100% acetonitrile. Then 100% acetonitrile was maintained for 2 min, followed by a linear gradient over 2 min to 5% acetonitrile/1,2-dichloroethane. Final conditions were held for 2 min before returning to initial conditions. Injection volume of all samples was 30 μl for the analysis. ROL eluted at 4.0 min, internal standard RE eluted at 7.9 min, and RE eluted at 16.5 min. Final amount of RA, ROL and total RE was normalized per million cells for cellular assays and per g tissue for skin analyses.

### RNA isolation and quantitative real-time PCR

For cells, total RNA was isolated from cultured keratinocytes using RNeasy Mini Kit (Qiagen, Valencia, CA, #74106) and treated with DNase I (Qiagen, #79254) to eliminate genomic DNA. For tissue, total RNA was isolated by following the manual of Direct-zol^TM^ RNA MiniPrep Plus kit (Zymo Research, R2073). The purity and concentration of RNAs were analyzed using a NanoDrop2000c (Thermo Fisher Scientific, ND-2000c). Following reverse transcriptase reactions using high-capacity RNA to cDNA kit (Life Technologies), qRT-PCR was performed to measure target genes using TaqMan probes and Fast reaction master mix reagents (Life Technologies). Relative expression of mRNAs was analyzed by the cycle of threshold (*C*_t_) value of target genes and quantified by normalizing to ribosomal protein large P0 (*RPLP0*) for human keratinocytes and to β-Actin for mouse keratinocytes or tissues as housekeeping genes using the ΔΔ*C*_t_ method^48^.

### Western blot analysis

Human and mouse keratinocytes were disrupted in M-PER lysis buffer (Thermo Fisher Scientific, #78501) containing protease inhibitors (Thermo Fisher Scientific, #87786) using ultrasonic homogenizer (20% power with 5 times every 2 s) to extract proteins. Then, protein concentrations were determined by BCA method (Thermo Fisher Scientific, #23225). Western blot procedures were followed by the protocol of NuPAGE system (Thermo Fisher Scientific). Briefly, 20 µg of protein samples were loaded for electrophoresis and transferred to polyvinylidene di-fluoride (PVDF) membrane (Bio-Rad, Hercules, CA). After blocking for at least an hour in 5% nonfat dry milk, the membrane was incubated with primary antibodies with appropriate dilutions (Supplementary table 2) at 4 °C overnight and followed by incubation with secondary antibodies for 1 h at room temperature. Protein amounts were normalized to rabbit polyclonal anti-human β-actin antibody (1:1000 dilution) (Cell Signaling Technology). Finally, proteins were visualized using SuperSignal^TM^ West Pico PLUS chemiluminescent substrate kit (Thermo Fisher Scientific, #34577) and saved as image files using ChemiDoc XRS^+^ (Bio-Rad). The signal intensity of protein was quantified using Image Lab^TM^ software (Bio-Rad). Original images of representative blots are provided in the Source data file.

### Immunofluorescence

For tissues, frozen-sections of mouse skin samples were stained with primary antibodies with appropriate dilution at 4 °C overnight, followed by incubation with goat anti-rabbit IgG labeled with Alexa Flour^®^ 488 (Life Technologies) and 594 (Life Technologies) for 1 h at room temperature. For cells, keratinocytes (3 × 10^5^ cells) plated on cover-slip were fixed with 4% Paraformaldehyde (PFA) for 10 min, permeabilized in PBS including 0.5% Triton X-100 for 15 min at room temperature. After washing with PBS 3 times, cells were stained with primary antibodies overnight at 4 °C, followed by incubation with goat anti-rabbit or mouse IgG labeled with Alexa Flour^®^ 488 for 1 h at room temperature. Cell nuclei were visualized by mounting medium including DAPI (Vector Laboratories, Burlingame, CA, H-1200). Fluorescence images were observed by fluorescence microscopy (Leica microsystems), taken pictures with ×100 (low magnification) and ×200 (high magnification), and saved as image files.

### Statistics

All data were created by at least three individual experiments to achieve statistical significances. For two groups, results were analyzed by Student *t*-test (unpaired or paired) to evaluate substantial differences using Microsoft Excel program. For more than three groups, the means of results were analyzed by one-way ANOVA using GraphPad Prism8 software. Data are represented as mean ± s.e.m. Statistical significance was obtained at *P*-value less than 0.05.

## Supplementary information

Supplementary Information

Source Data

## Data Availability

The authors declare that data supporting the findings of this study are available within the article and its supplementary information files or from the corresponding author upon reasonable request. Data for microarray analyses have been deposited in the Gene Expression Omnibus (GEO) database under accession codes GSE128868 and GSE131789. All proteomic datasets are available to download from the data repository of University of Maryland metallotherapeutics research center, Baltimore [https://bit.ly/2U9qwqO] and the mass spectrometry proteomics data have been deposited to the ProteomeXchange Consortium via the PRIDE partner repository with the dataset identifier PXD013854. All raw images of western blot for Figs. 1g, 3a, 3b, 4b, 4d, 4h, 5a and 5b are provided in the Source data file.
