# Supplementary material for: Noncoding dsRNA induces retinoic acid synthesis to stimulate hair follicle regeneration via TLR3
Source: Nat Commun. 2019 Jun 26;10:2811. doi: 10.1038/s41467-019-10811-y (PMC6594970; doi:10.1038/s41467-019-10811-y)
Supplement: Supplementary file 1 — Supplementary Information [file 41467_2019_10811_MOESM1_ESM.pdf]

Supplementary Information

- Supplementary Figures and Legends
- Supplementary Tables

Noncoding dsRNA induces retinoic acid synthesis to stimulate hair follicle regeneration via TLR3

Kim, et al.

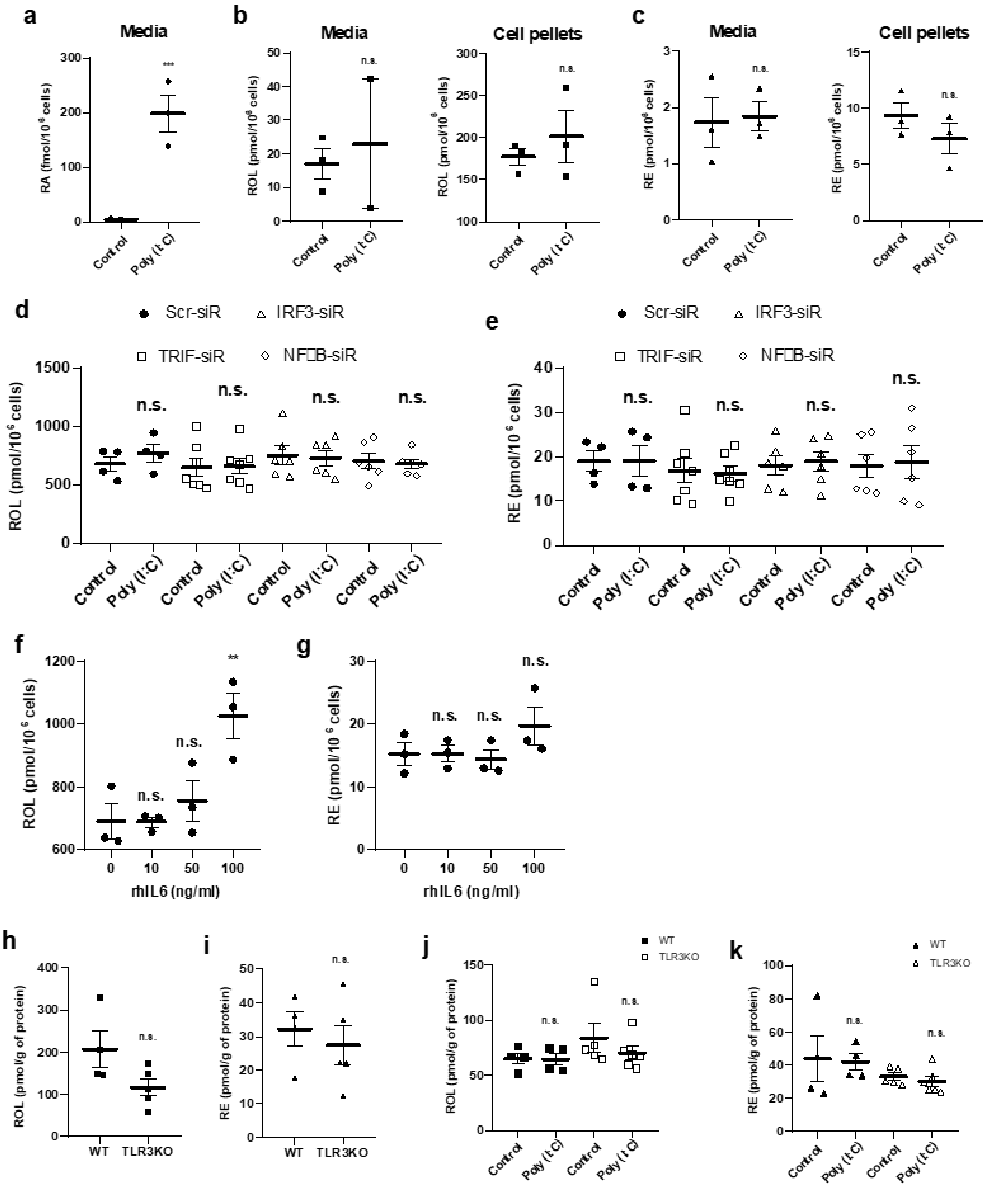

a
Media
RA (fmol/10⁶ cells)
***
Control
Poly (I:C)
b
Media
ROL (pmol/10⁶ cells)
n.s.
Cell pellets
ROL (pmol/10⁶ cells)
n.s.
c
Media
RE (pmol/10⁶ cells)
n.s.
Cell pellets
RE (pmol/10⁶ cells)
n.s.
d
Scr-siR
IRF3-siR
TRIF-siR
NF□B-siR
ROL (pmol/10⁶ cells)
n.s.
e
RE (pmol/10⁶ cells)
n.s.
f
ROL (pmol/10⁶ cells)
n.s.
**
rhIL6 (ng/ml)
g
RE (pmol/10⁶ cells)
n.s.
rhIL6 (ng/ml)
h
ROL (pmol/g of protein)
n.s.
WT
TLR3KO
i
RE (pmol/g of protein)
n.s.
j
WT
TLR3KO
ROL (pmol/g of protein)
n.s.
k
WT
TLR3KO
RE (pmol/g of protein)
n.s.

Supplementary Figure 1. *In vitro* and *in vivo* analysis of vitamin A metabolites using LC-MS

a, Analysis of RA in cultured media in human keratinocytes treated with PBS (control) and Poly (I:C) (n=3 independent experiments, ***$P<0.001$, paired student *t*-test). **b**, Analysis of retinol (ROL) in cultured media and cell lysates in human keratinocytes treated with PBS (control) and Poly (I:C) (n=2-3 independent experiments). n.s.; not significant. **c**, Analysis of retinyl ester (RE) in cultured media and cell lysates in human keratinocytes treated with PBS (control) and Poly (I:C) (n=3 independent experiments). n.s.; not significant. **d-e**, Analysis of ROL (d) and RE (e) in human keratinocytes transfected with TLR3 downstream targets in presence of PBS (control) and Poly (I:C) (n=4-6 independent experiments). n.s.; not significant. **f-g**, Analysis of ROL (f) and RE (g) in human keratinocytes treated with recombinant human IL6 (n=3 independent experiments, **$P<0.01$, on-way ANOVA) **h-i**, Analysis of ROL (h) and RE (i) in unwounded skin of WT and $Tlr3^{-/-}$ mice (n=4 independent animals). n.s.; not significant. **j-k**, Analysis of ROL (j) and RE (k) in wounded skin of WT and $Tlr3^{-/-}$ mice treated with PBS (control) and Poly (I:C) (n=4-5 independent animals). n.s.; not significant. Data are means±SEM.

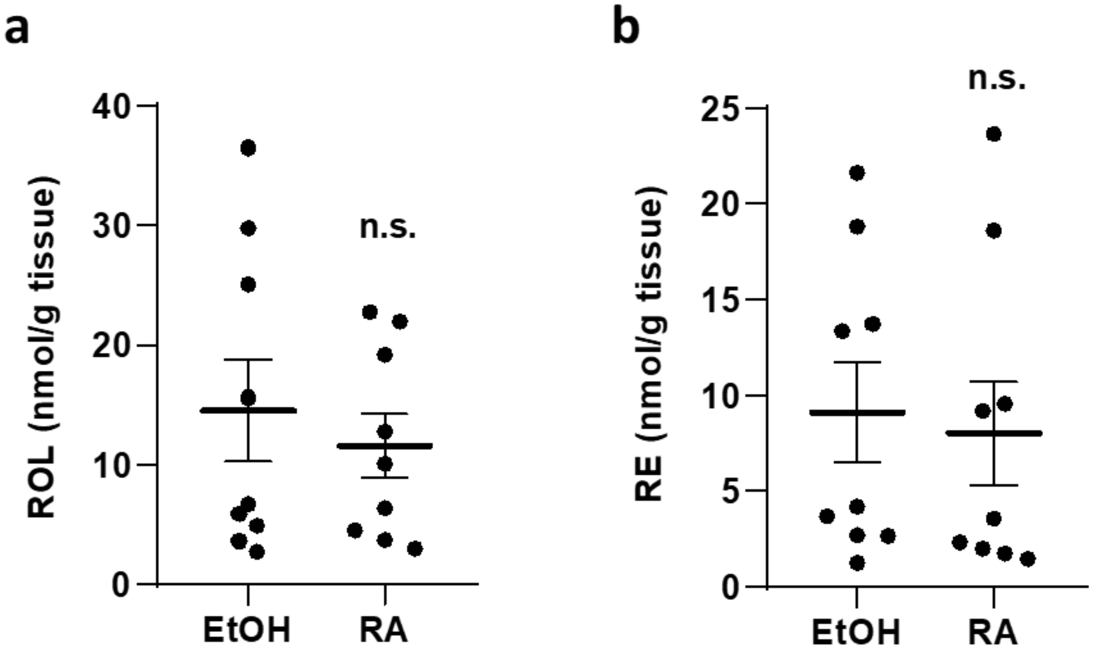

Supplementary Figure 2. *In vivo* analysis of vitamin A metabolites in *Tlr3*$^{-/-}$ mice with exogenous RA treatment using LC-MS

a-b, Analysis of ROL (a) and RE (b) in wounded skin of WT and *Tlr3*$^{-/-}$ mice with treatment of Ethanol (EtOH) and RA. (n=9 independent animals). n.s.; not significant. Data are means±SEM.

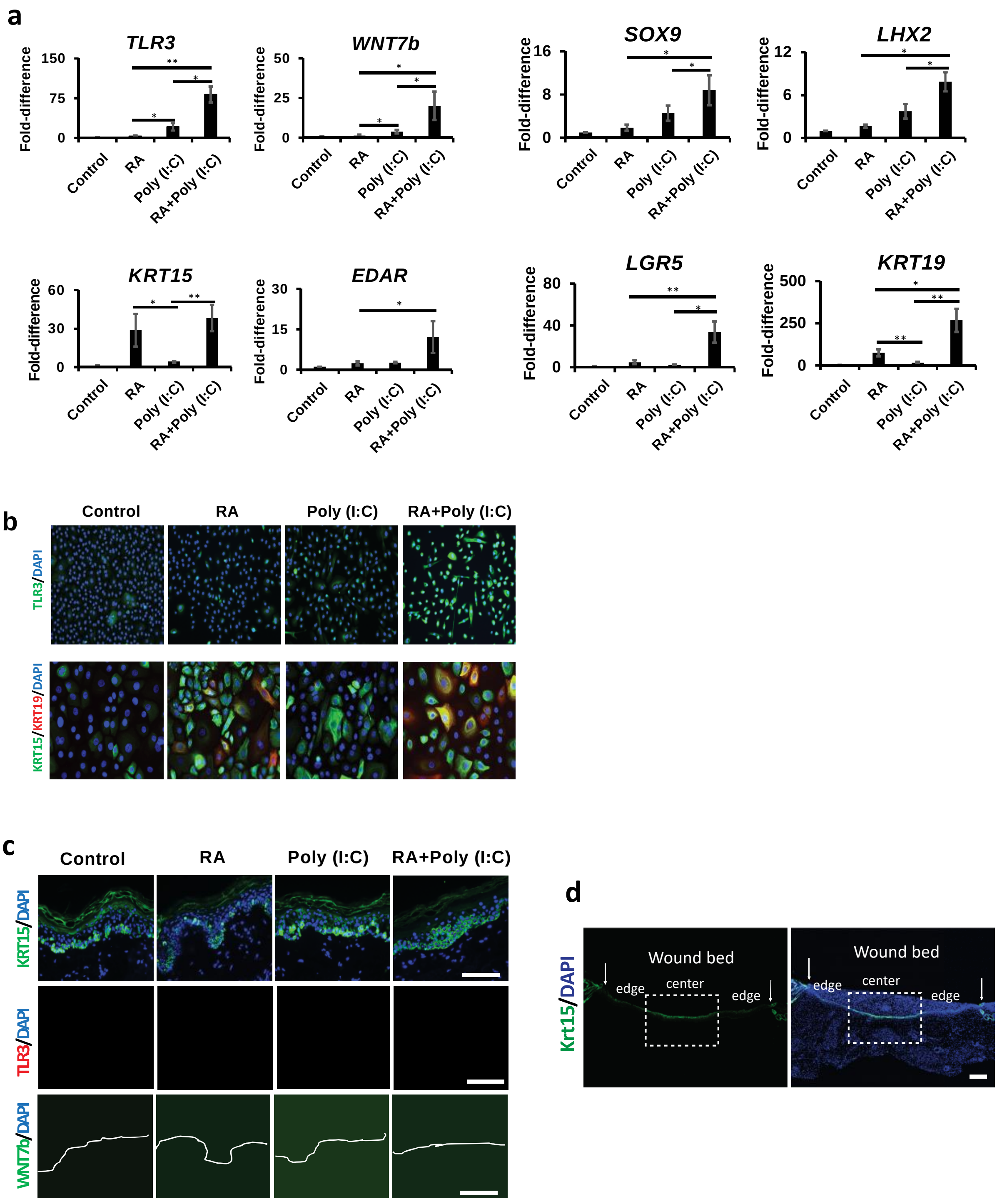

Supplementary Figure 3. Synergistic effects of RA and Poly (I:C)

a, Relative mRNA expression of *TLR3*, *WNT7b*, *SOX9*, *LHX2*, *KRT15*, *EDAR*, *LGR5*, and *KRT19* in human scalp keratinocytes with RA and/or Poly (I:C). (n=5 independent experiments,

*P<0.05 and **P<0.01, one-way ANOVA). **b**, Immunostaining results of TLR3 (green), KRT15 (green), and KRT19 (red) in human scalp keratinocytes with RA and/or Poly (I:C). Nucleus was stained by DAPI (blue). **c.** Human whole skin explants treated as above and stained for KRT15 (green), TLR3 (red) and WNT7b (green). White Scale bar: 100 µm. Note suprabasal staining of KRT15/TLR3 and confluent basal layer staining of WNT7b in combination treatment. **d**, Immunostaining results of Krt15 (green) in wounded skin of WT mice at Day 11 after wounding. Nucleus was stained by DAPI (blue). White arrow indicates the edge between wounded and unwounded skin. White dashed line indicates areas with new hair follicles. White Scale bar: 100 µm. Data are means±SEM.

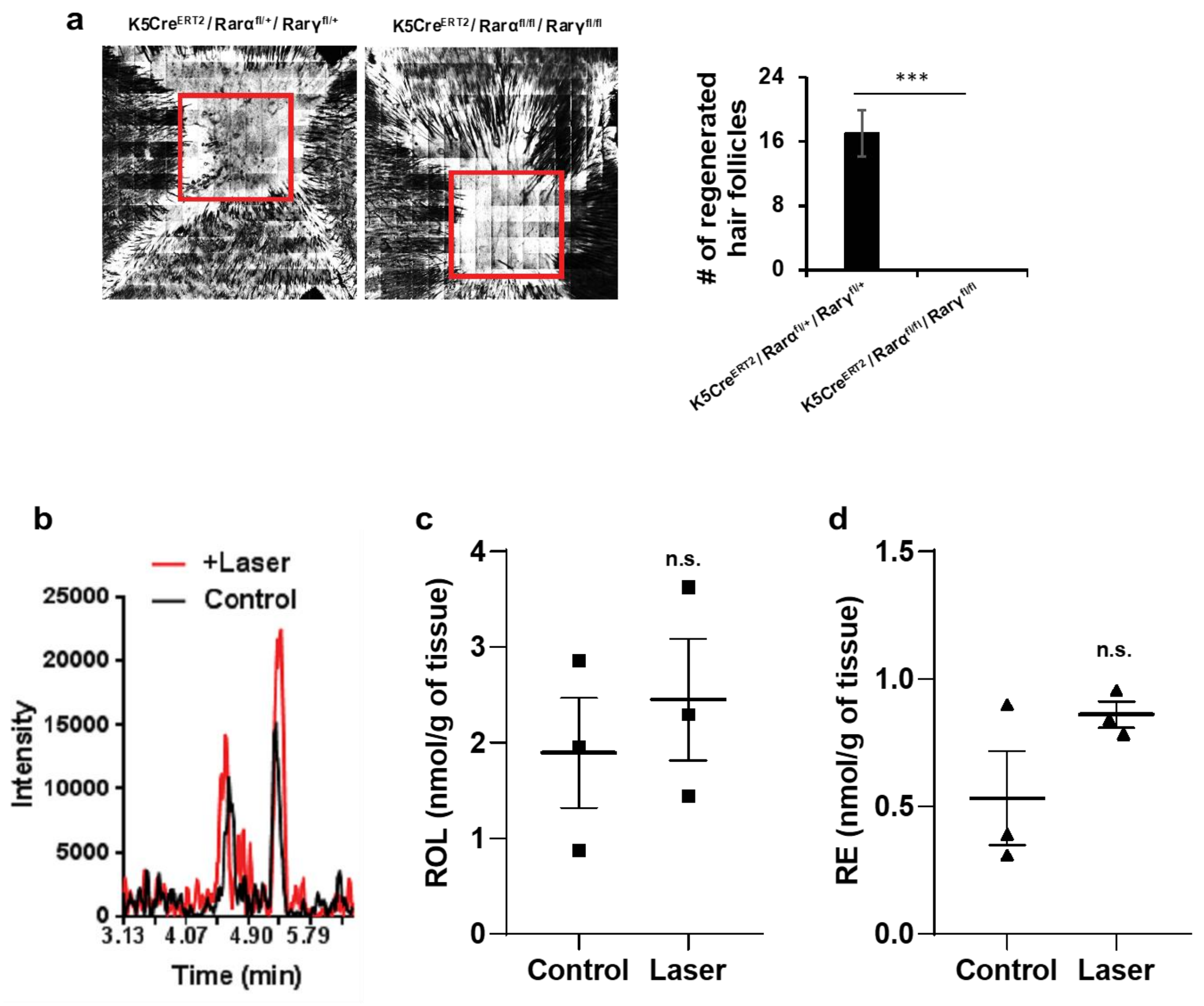

Supplementary Figure 4. WIHN in Rar-alpha and gamma double knockout mice and analysis of vitamin A metabolites in human subjects

a, Skin specific Rara and Rarg double knockout mice fail to regenerate hair follicles (n=4 independent animals, ****P*<0.001, paired student *t*-test). Red square indicates areas with new hair follicles. **b**, Intensity of RA in human subjects with CO_2 laser treatment. **c-d**, Analysis of ROL (c) and RE (d) in human subjects with CO_2 laser treatment. (n=3 independent human samples), n.s.; not significant. Data are means±SEM.

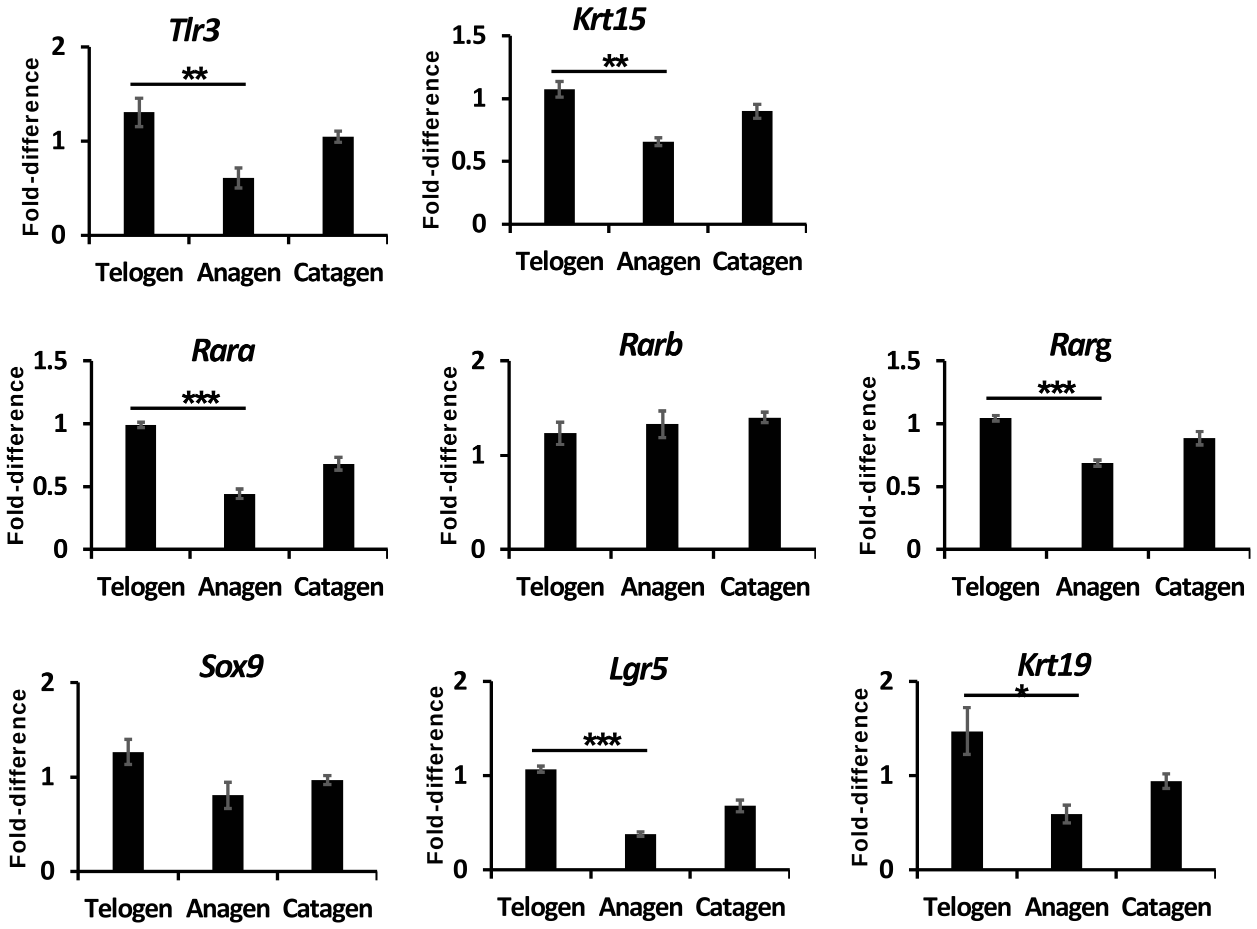

Supplementary Figure 5. Gene expression during the hair cycle

a, Relative mRNA expression of *Tlr3, Krt15, Rara, Rarb, Rarg, Sox9, Lgr5,* and *Krt19* in Krt5-Cre mouse skin during the hair cycle. (n=4-7 independent animals, *P<0.05, **P<0.01, ***P<0.05, unpaired student t-test). Note coordinated expression of tested genes during the quiescent telogen phase. Data are means±SEM.

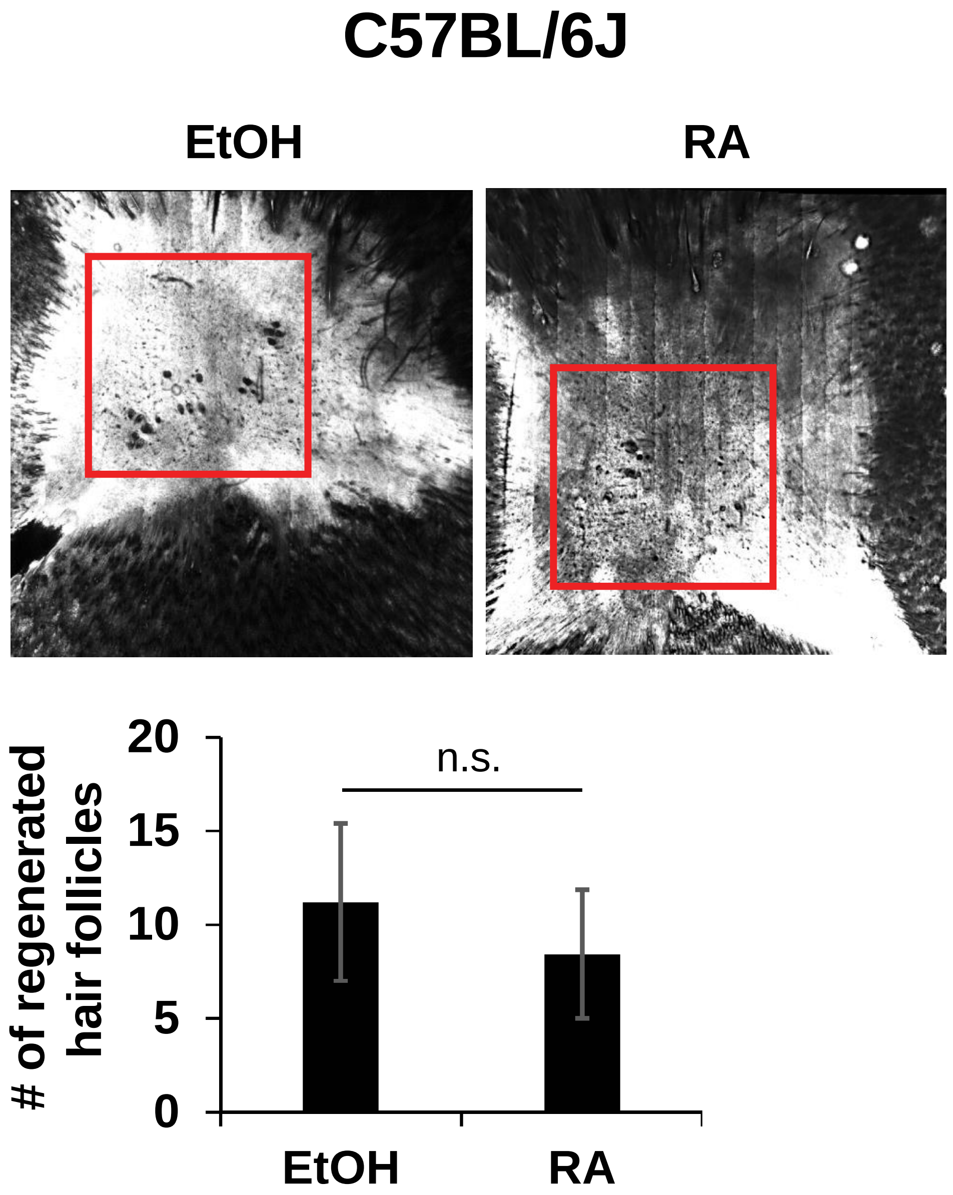

Supplementary Figure 6. Exogenous RA treatment in WT mice

Top, Confocal image of WT wounded skin with ethanol (EtOH) and RA treatment. **Bottom**, Quantified WIHN results (EtOH; n=5, RA; n=9 independent animals). Red square indicates areas with new hair follicles. n.s.; not significant. Data are means±SEM.

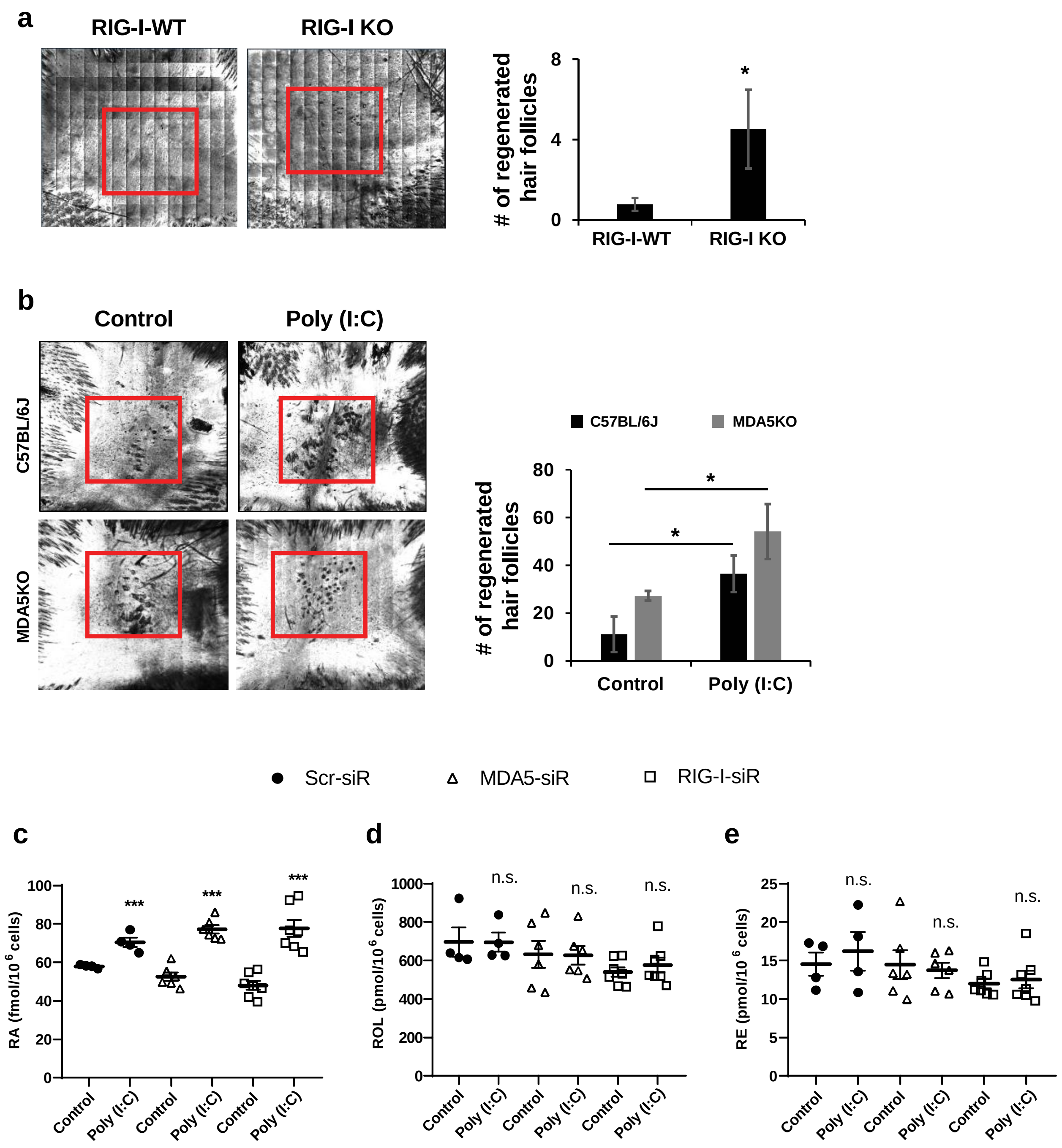

Supplementary Figure 7. Hair follicle regeneration, Vitamin A, and its metabolites in *MDA5*$^{-/-}$ and *RIG-I*$^{-/-}$ mice

a, WIHN results of strain-matched WT (n=9 independent animals) and *RIG-I*$^{-/-}$ (n=17 independent animals) mice (*P<0.05, unpaired student *t*-test). Red square indicates areas with

new hair follicles. **b**, WIHN results of WT and *MDA5*$^{-/-}$ mice treated with PBS (control) and Poly (I:C) (n=4-5 independent animals, *$P<0.05$, unpaired student *t*-test). Note that Poly (I:C) induces hair follicle regeneration in *MDA5*$^{-/-}$ mice similar to WT. **c-e**, analysis of RA (c), ROL (d), and RE (e) in human keratinocytes transfected with scrambled-, MDA5-, and RIG-I-siRNA in presence of PBS (Control) and Poly (I:C) (n=4-7 independent experiments, ***$P<0.001$, one-way ANOVA), n.s.; not significant. Data are means±SEM.

Supplementary Table 1. Primer sets for genotyping

Name		Sequences (5’-3’)	Product size (bp)
Cre	Forward	GAC CAG GTT CGT TCA CTC ATG G	200
	Reverse	AGG CTA AGT GCC TTC TCT ACA C	
Rarα flox	Forward	CAG GGA GGA TGC TGT TTG TA	WT; 156
	Reverse	CCT ATG ACC CAG GAC TCA GC	Mut: 189
Rarɣ flox	Forward	AGC TCA GTG GTG GAA TGC TT	WT; 240
	Reverse	TTT TCT GAA TGC TGC GTC TG	Mut: 280
Rarα KO	Forward	TTT GCC TGC TCT TCT GAC TG	
	Reverse (WT)	ACG GTG TGC TGT AAC CAC TG	WT; 201
	Reverse (Mut)	GCC AGA GGC CAC TTG TGT AG	Mut; 165
Tlr3 KO	Forward	AAT TCA TCA GTG CCA TGA GTT T	
	Reverse (WT)	GCA ACC CTT TCA AAA ACC AG	WT; 341
	Reverse (Mut)	GCC AGA GGC CAC TTG TGT AG	Mut; 208

Supplementary Table 2. **Antibodies used for western blot and immunostaining**

Name	Host	Dilution		Company/Cat No.
		Western blot	Immunostaining	
TLR3	mouse		1:300	Santa Cruz Biotechnology #sc-32232
KRT19	Mouse	1:2,000	1:300	Invitrogen, #MA5-12663
KRT15	Rabbit	1:2,000	1:300	Sigma, HPA023910
ALDH1A3	Rabbit	1:1,000		Invitrogen, #PA5-61201
β-Actin	Rabbit	1:1,000		Cell Signaling Technology, #4967L
β-catenin	mouse	1:1,000		Millipore, 610153
Active β-catenin	mouse	1:1,000		Millipore, #05-665/clone 8E7
WNT7b	Rabbit		1:200	Abcam, #ab94915
Alexa Fluor® 488 Anti-Rabbit IgG (H+L)	Goat		1:1,000	Life Technologies, A-11034
Alexa Fluor® 594 Anti-Rabbit IgG (H+L)	Goat		1:1,000	Life Technologies, A-11037
anti-mouse IgG-HRP		1:1,000		Cell Signaling Technology; #7076S
anti-rabbit IgG-HRP		1:1,000		Cell Signaling Technology; #7074S

Supplementary Table 3. Mouse type, strain, chemical, vehicle, schedule for *in vivo* experiments

Mouse	Mouse strain	Treatment	Vehicle	Day of treatment	Note
Wild type (WT)	C57BL/6J	Poly (I:C)	PBS	WD3	JAX stock #00664
		BMS493	DMSO	WD1	
Wild type (WT)	C57BL/6NJ	Poly (I:C)	PBS	WD3	JAX stock #005304<br>Used for Tlr3-/-
		RA	Ethanol	WD1	
Tlr3$^{-/-}$	B6N.129S1-Tlr3$^{tm1Flv/}$J	Poly (I:C)	PBS	WD3	JAX stock #009675
		RA	Ethanol	WD1	
Mda5$^{-/-}$	B6.Cg-Ifih1$^{tm1.1Cln}$/J	Poly (I:C)	PBS	WD3	JAX stock #015812
Rigi$^{-/-}$	129Sv/C57BL/6-Ddx58				No treatment
Rigi-WT	129Sv/C57BL/6				No treatment
Rarα$^{-/-}$	Rara^{tm1Rev}/HsvJ	Poly (I:C)	PBS	WD3	JAX stock #023845
Krt5-CreERT2	C57BL/6J	Tamoxifen	Corn oil	D15 to WD3	Crossed with floxed mice
Rarα$^{fl/fl}$	C57BL/6J-Rarα floxed	Tamoxifen	Corn oil	D15 to WD3	Crossed with Krt5-CreERT2 mice
Rarγ$^{fl/fl}$	C57BL/6J-Rarγ floxed	Tamoxifen	Corn oil	D15 to WD3	
Rarα-Rarγ$^{fl/fl}$	C57BL/6J-Rarα-Rarγ floxed (double floxed)	Tamoxifen	Corn oil	D15 to WD3	

D15: 15th day after born, WD3: 3rd day after wounding
