# Supplementary figures and images for: Noncoding dsRNA induces retinoic acid synthesis to stimulate hair follicle regeneration via TLR3
Source: Nat Commun. 2019 Jun 26;10:2811. doi: 10.1038/s41467-019-10811-y (PMC6594970; doi:10.1038/s41467-019-10811-y)

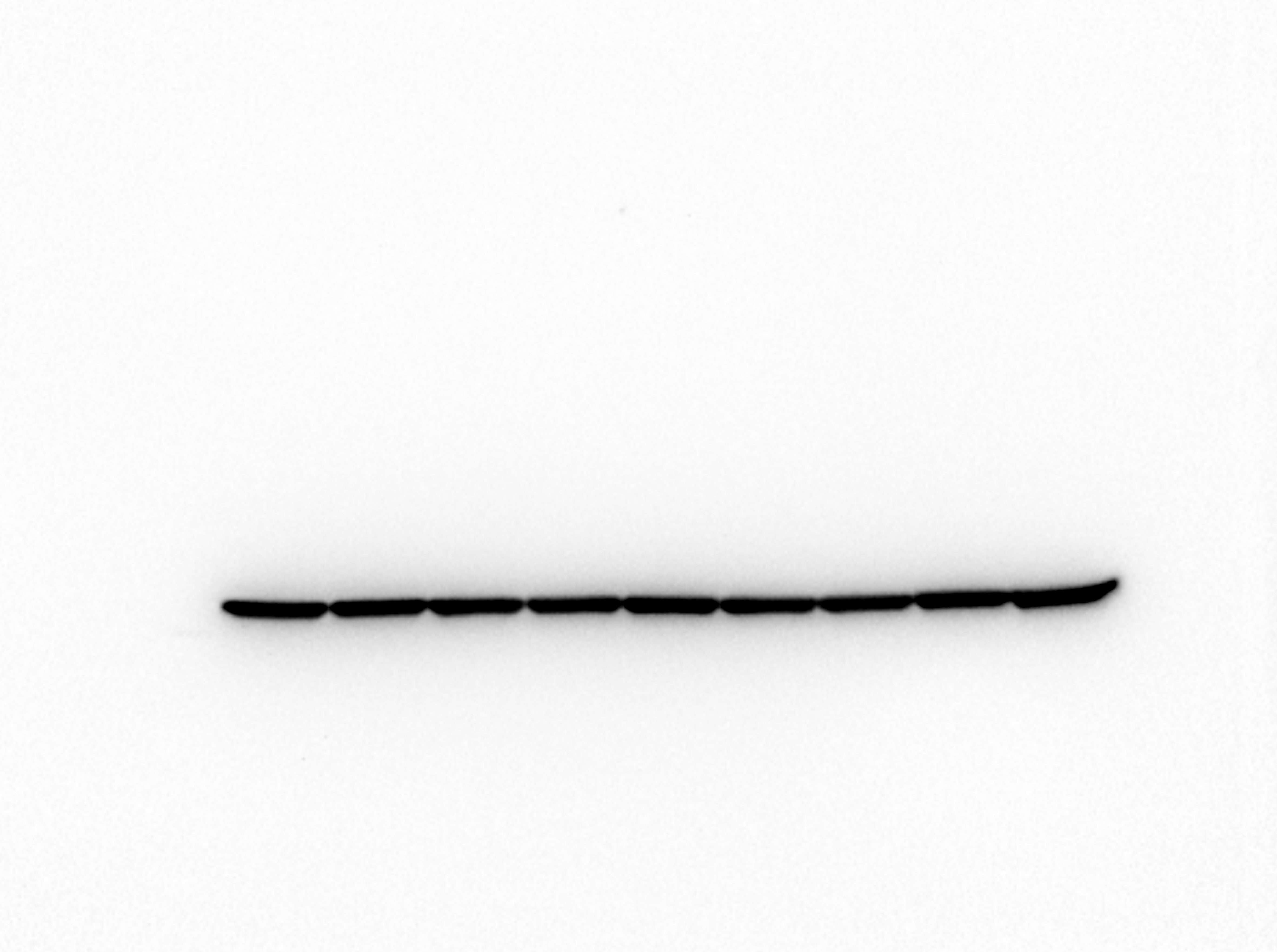

Supplement: Supplementary file 2 — Source Data [file 41467_2019_10811_MOESM2_ESM.zip › the Source Data file/Figure 1g/bActin for K19_Biolog 2018-12-02 14hr 58min_Exposure_20.0sec.jpg]

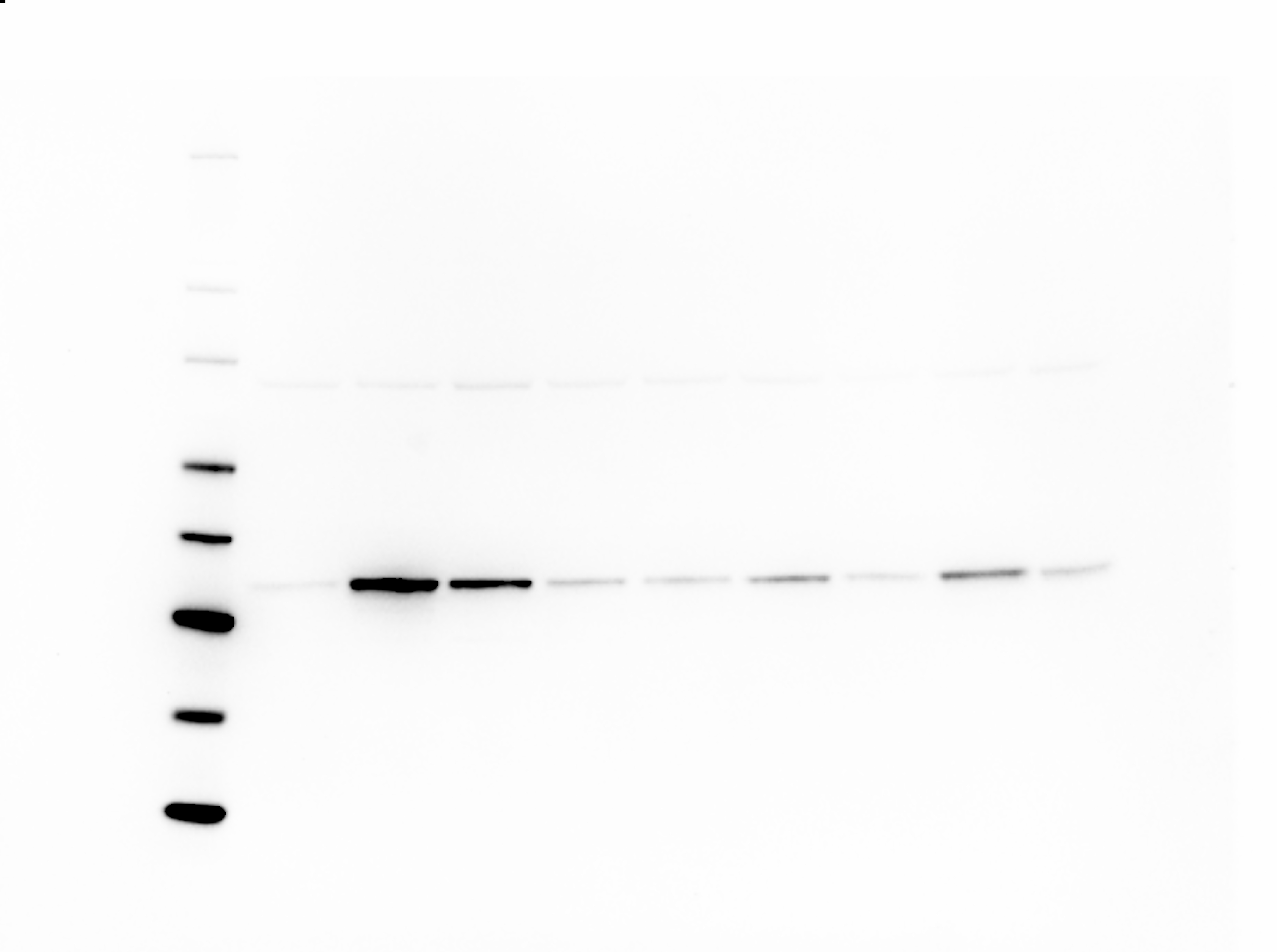

Supplement: Supplementary file 2 — Source Data [file 41467_2019_10811_MOESM2_ESM.zip › the Source Data file/Figure 1g/KRT19 in TLR3 and RARg-siR-Biolog 2018-11-29 12hr 34min_Exposure_20.0sec.jpg]

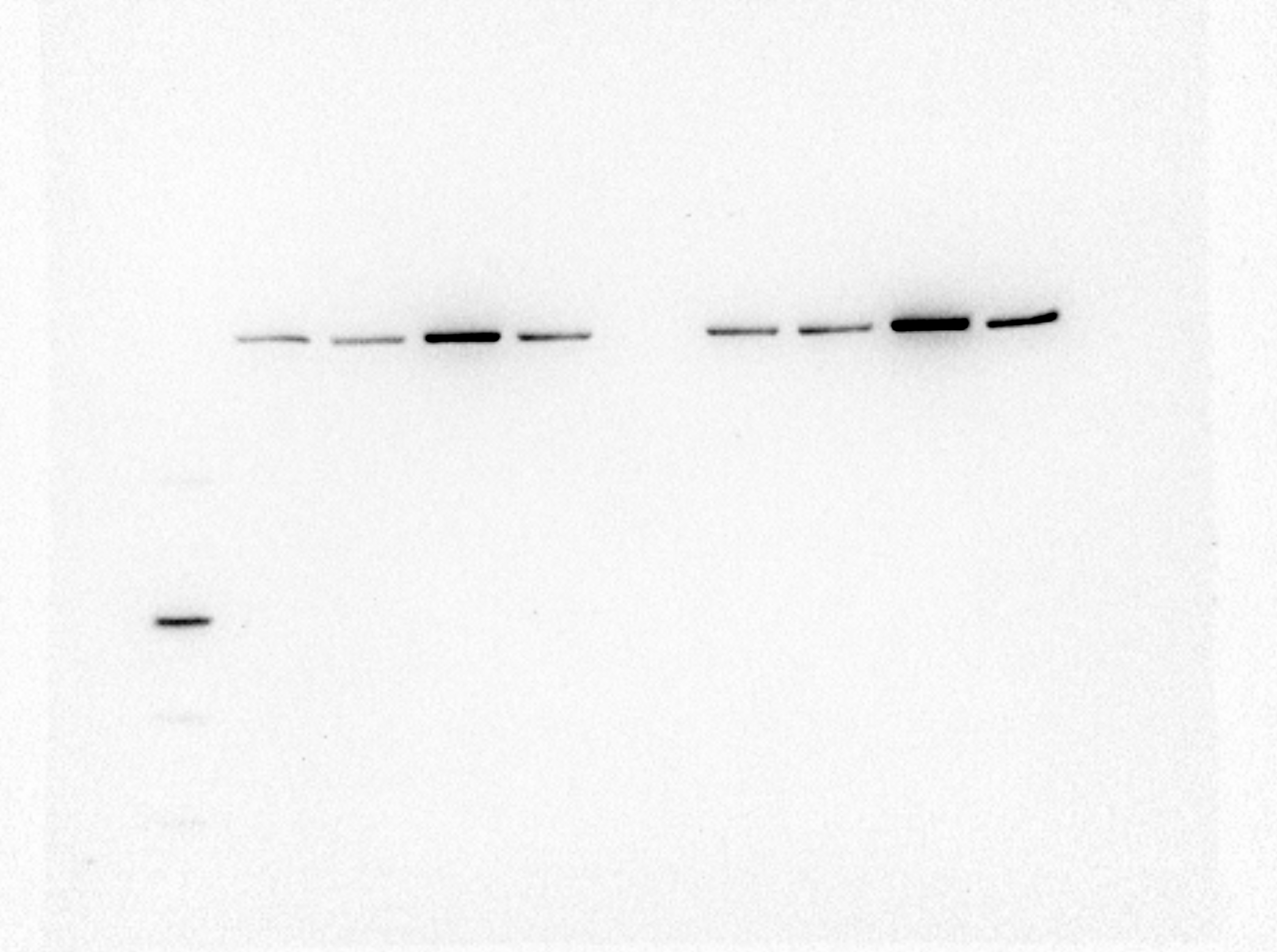

Supplement: Supplementary file 2 — Source Data [file 41467_2019_10811_MOESM2_ESM.zip › the Source Data file/Figure 3a/ABC-WB.jpg]

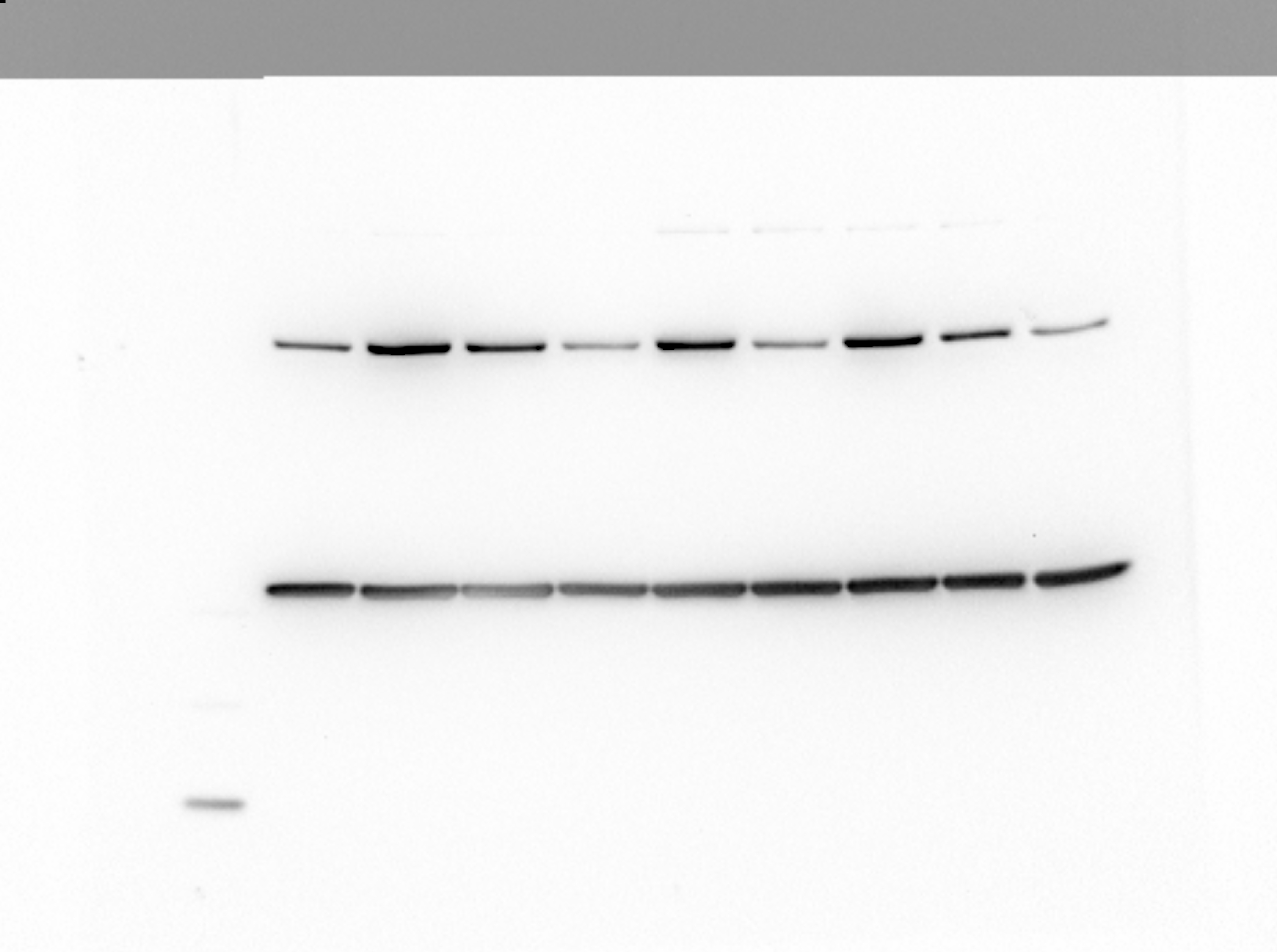

Supplement: Supplementary file 2 — Source Data [file 41467_2019_10811_MOESM2_ESM.zip › the Source Data file/Figure 3a/bActin for ABC and total catenin-WB.jpg]

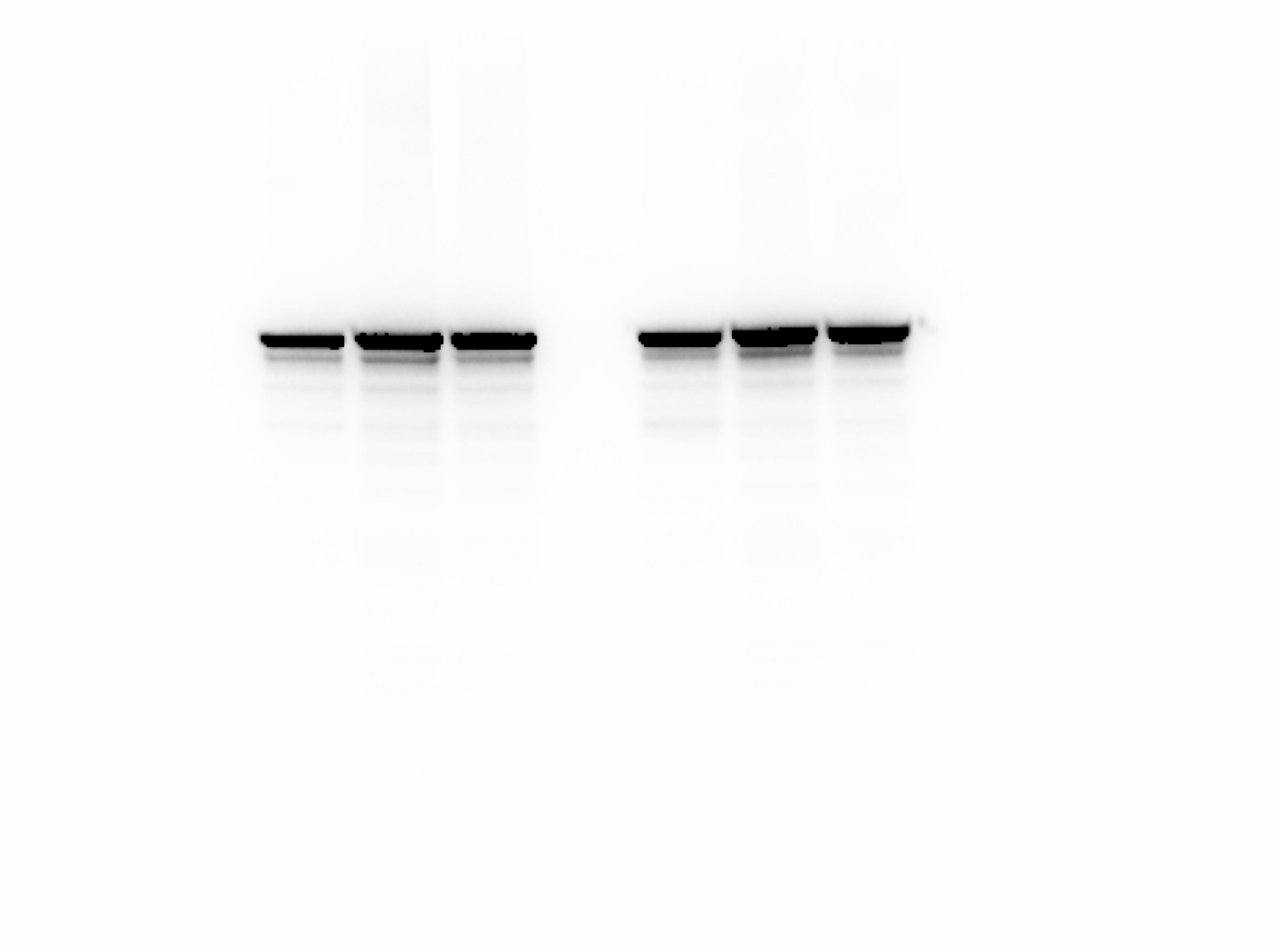

Supplement: Supplementary file 2 — Source Data [file 41467_2019_10811_MOESM2_ESM.zip › the Source Data file/Figure 3a/b-catenin-WB.jpg]

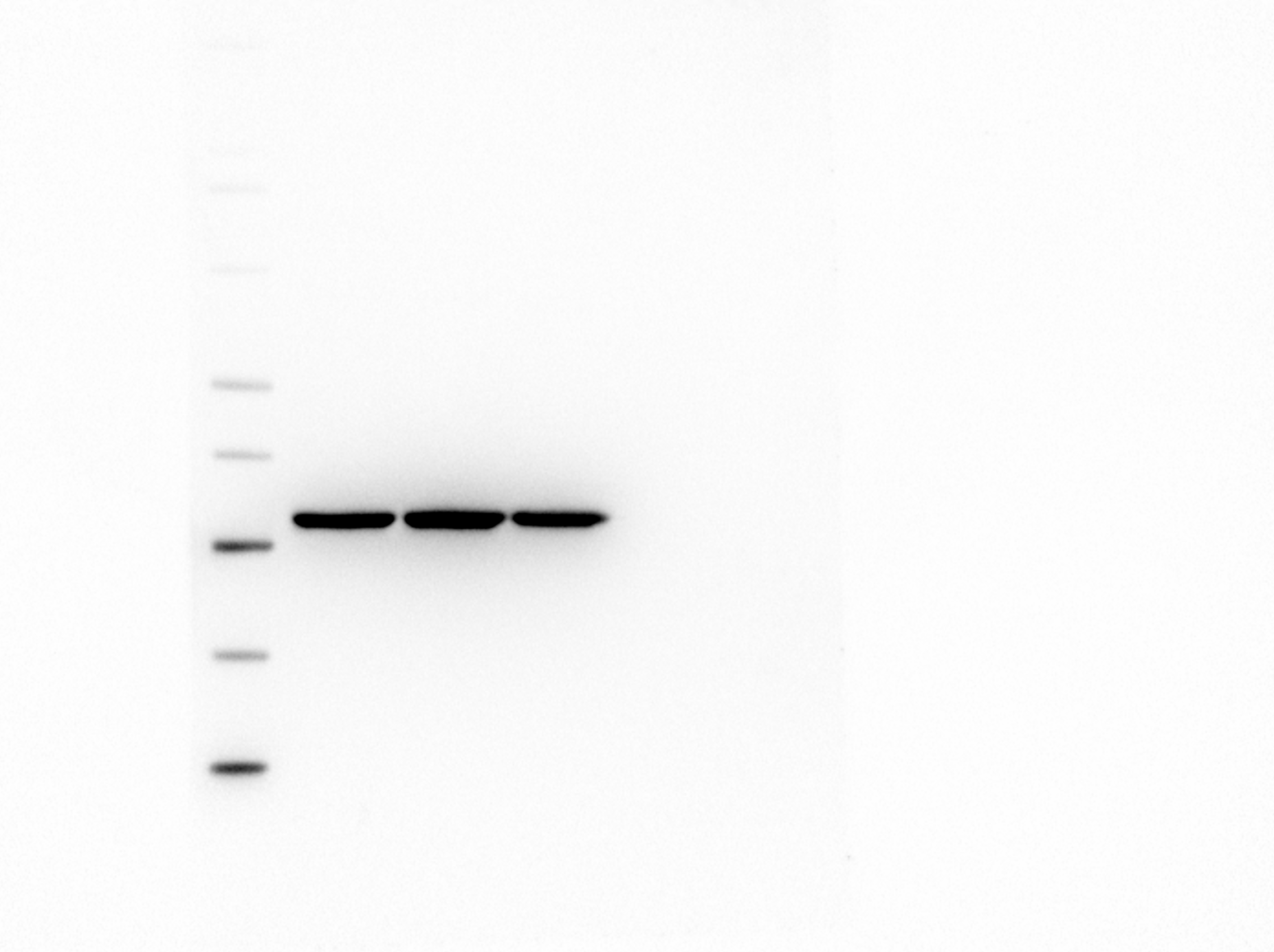

Supplement: Supplementary file 2 — Source Data [file 41467_2019_10811_MOESM2_ESM.zip › the Source Data file/Figure 3b/bActin for K15 K19 in TLR3KO kers-WB.jpg]

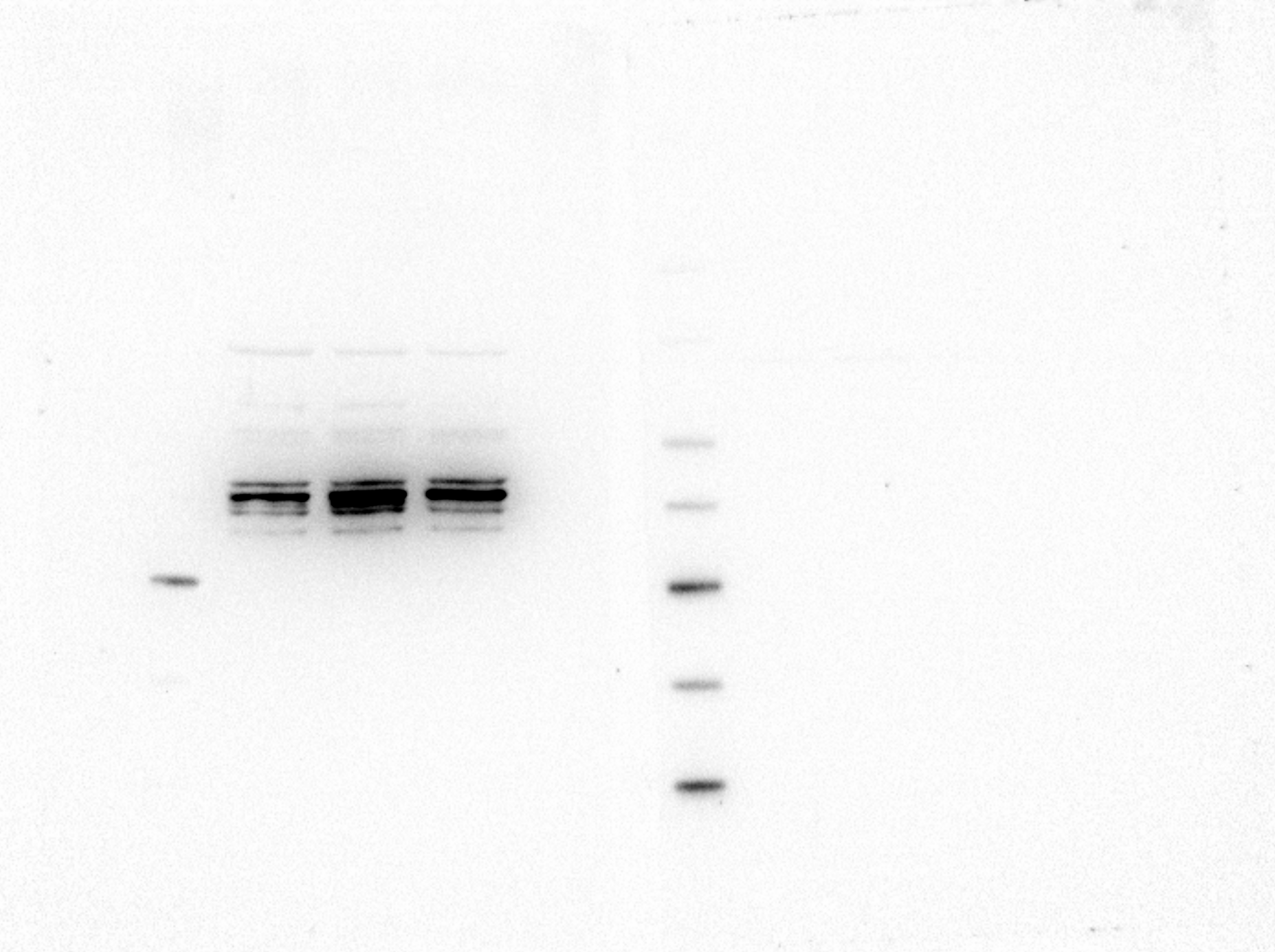

Supplement: Supplementary file 2 — Source Data [file 41467_2019_10811_MOESM2_ESM.zip › the Source Data file/Figure 3b/K15-TLR3KO kers-WB.jpg]

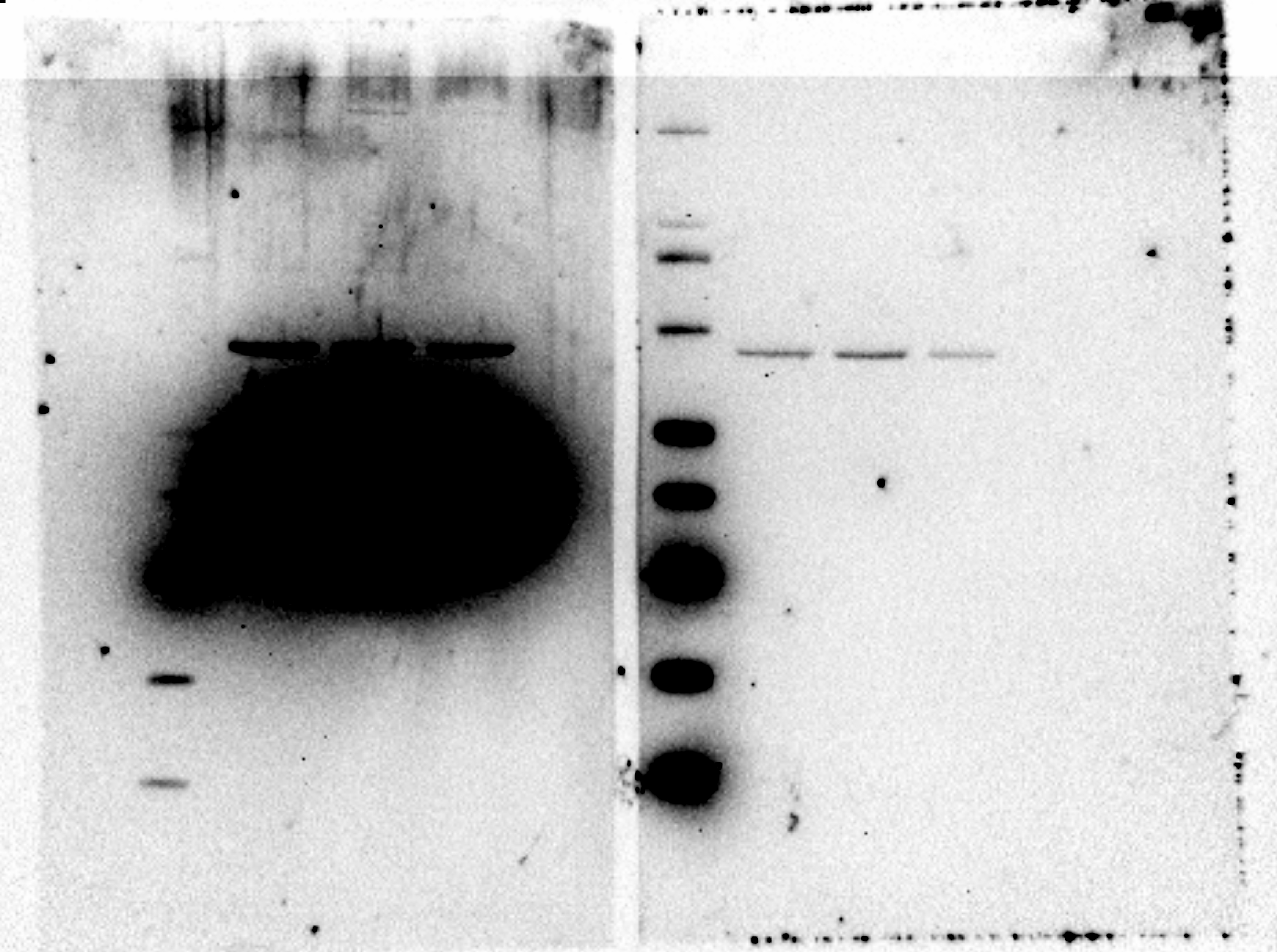

Supplement: Supplementary file 2 — Source Data [file 41467_2019_10811_MOESM2_ESM.zip › the Source Data file/Figure 3b/K19-TLR3KO kers-WB.jpg]

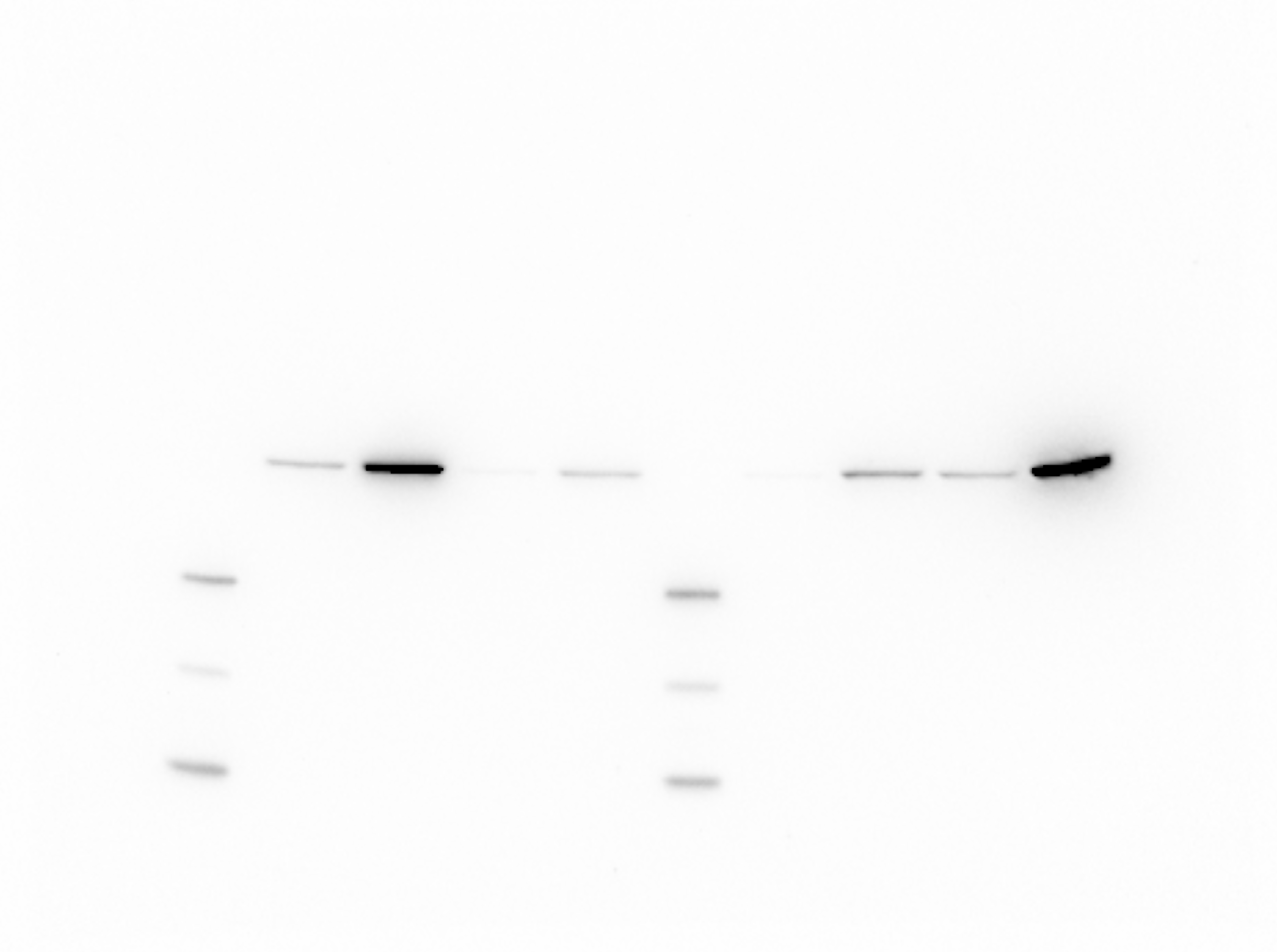

Supplement: Supplementary file 2 — Source Data [file 41467_2019_10811_MOESM2_ESM.zip › the Source Data file/Figure 4b/ALDH1A3 for siRNA and DEAB-WB.jpg]

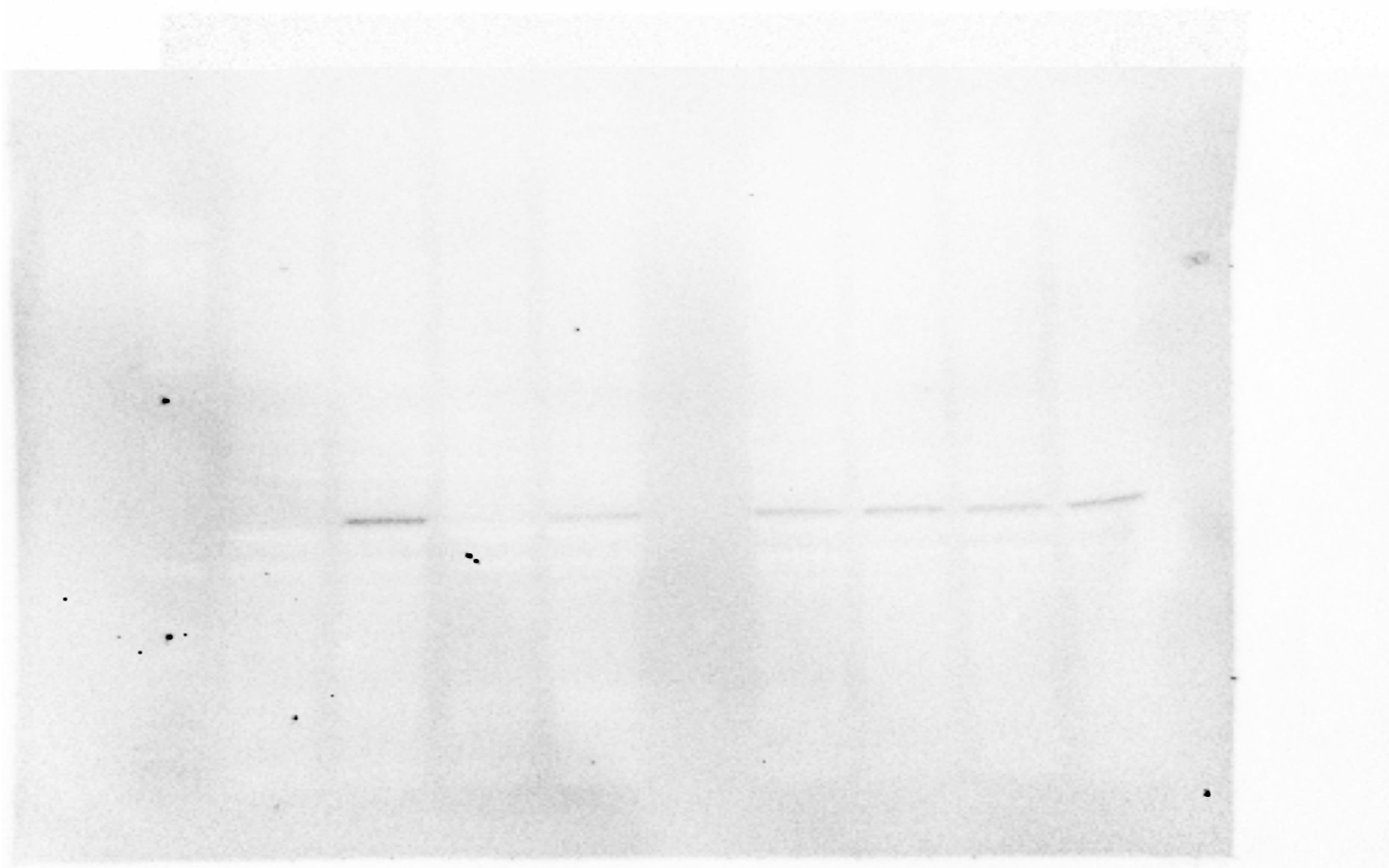

Supplement: Supplementary file 2 — Source Data [file 41467_2019_10811_MOESM2_ESM.zip › the Source Data file/Figure 4b/K19_A2-A3 siRNA with PIC-WB.jpg]

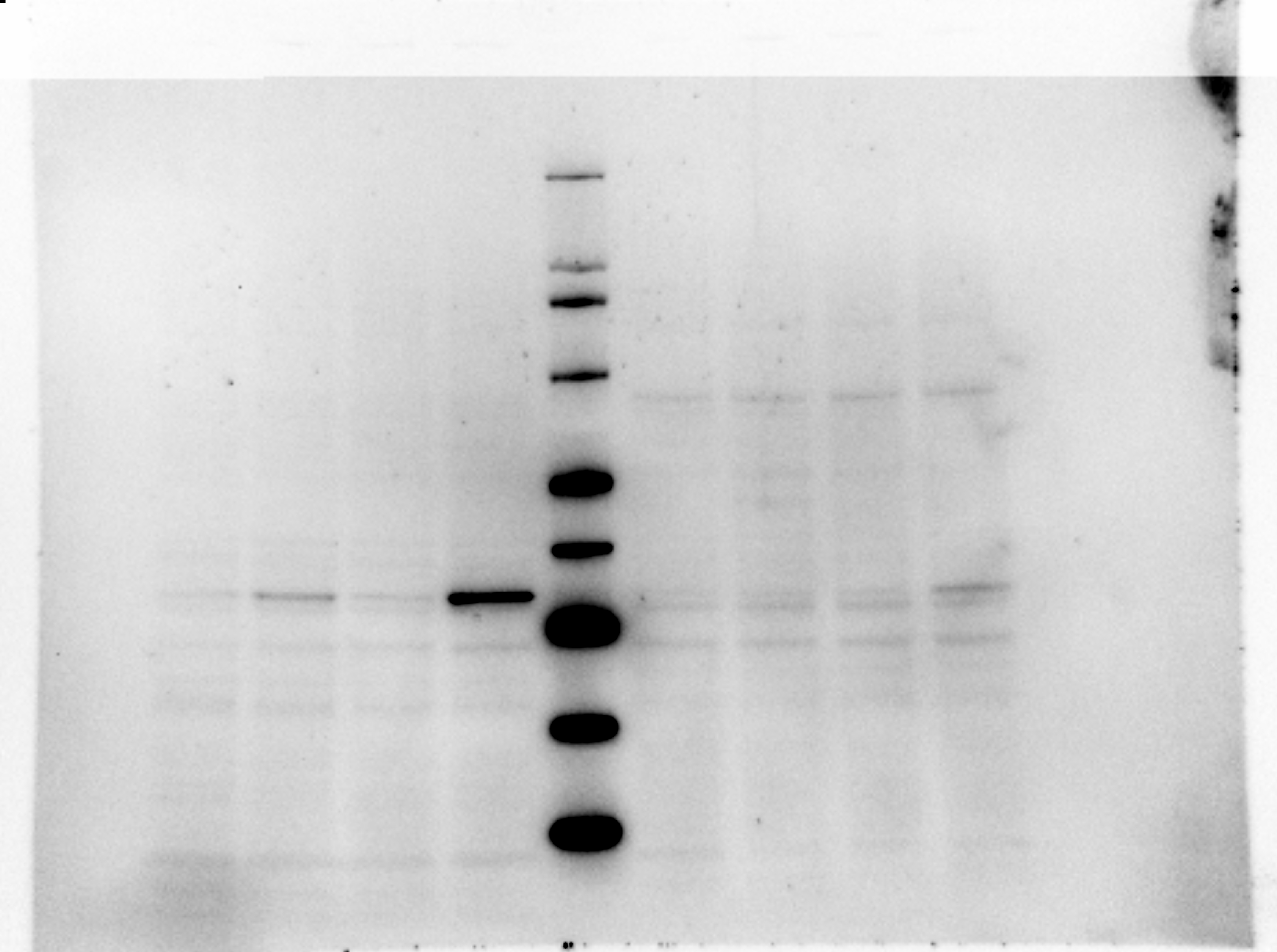

Supplement: Supplementary file 2 — Source Data [file 41467_2019_10811_MOESM2_ESM.zip › the Source Data file/Figure 4b/K19_DEAB with PIC-WB.jpg]

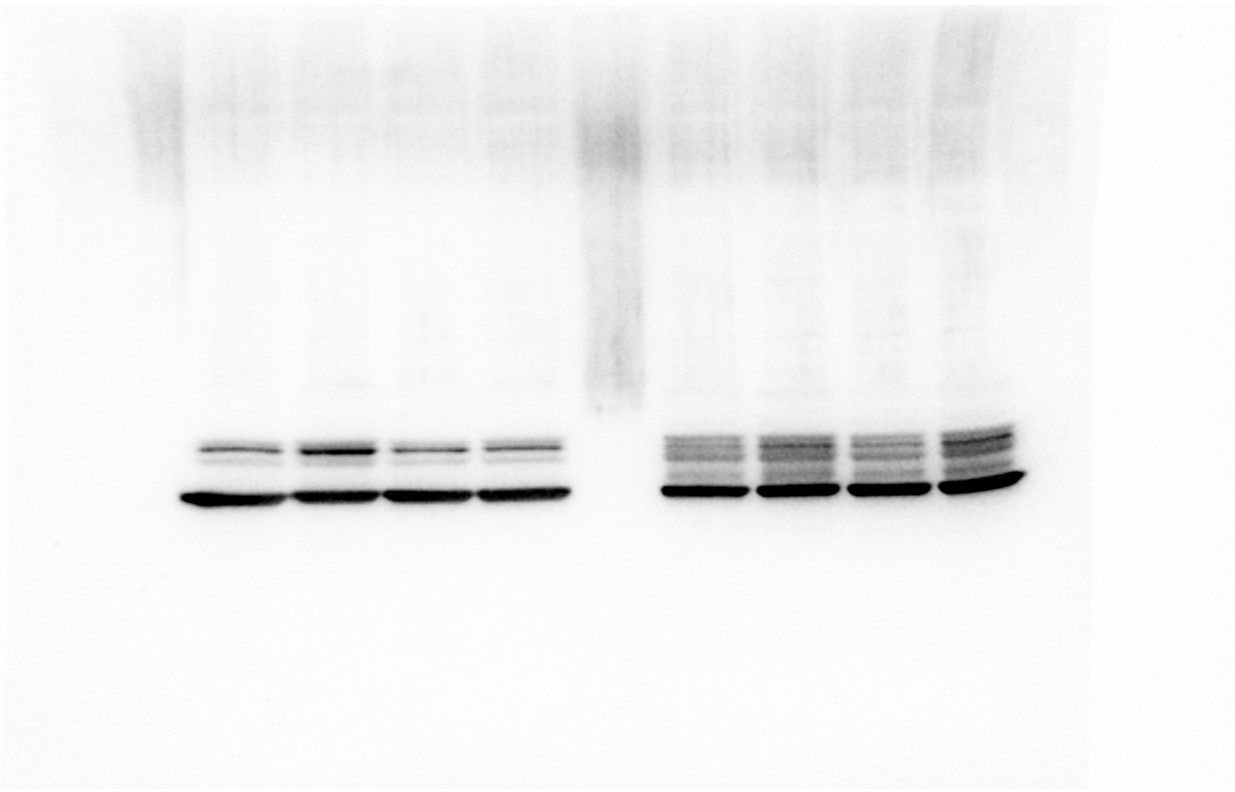

Supplement: Supplementary file 2 — Source Data [file 41467_2019_10811_MOESM2_ESM.zip › the Source Data file/Figure 4b/KRT15 and bActin-A2-A3-siRNA with PIC-WB.jpg]

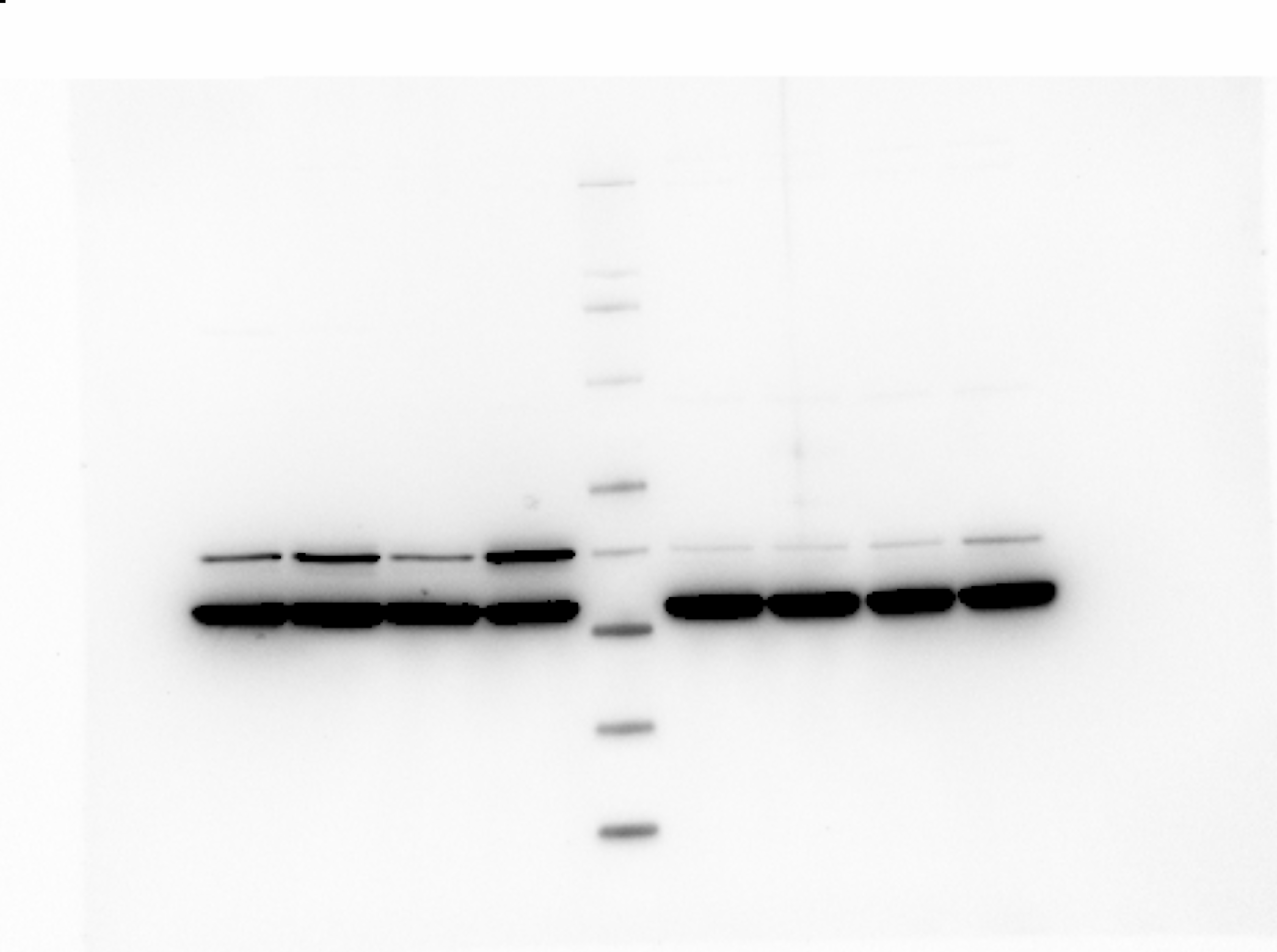

Supplement: Supplementary file 2 — Source Data [file 41467_2019_10811_MOESM2_ESM.zip › the Source Data file/Figure 4b/KRT15_bActin_DEAB_Biolog 2018-01-23 18hr 07min_Exposure_10.0sec.jpg]

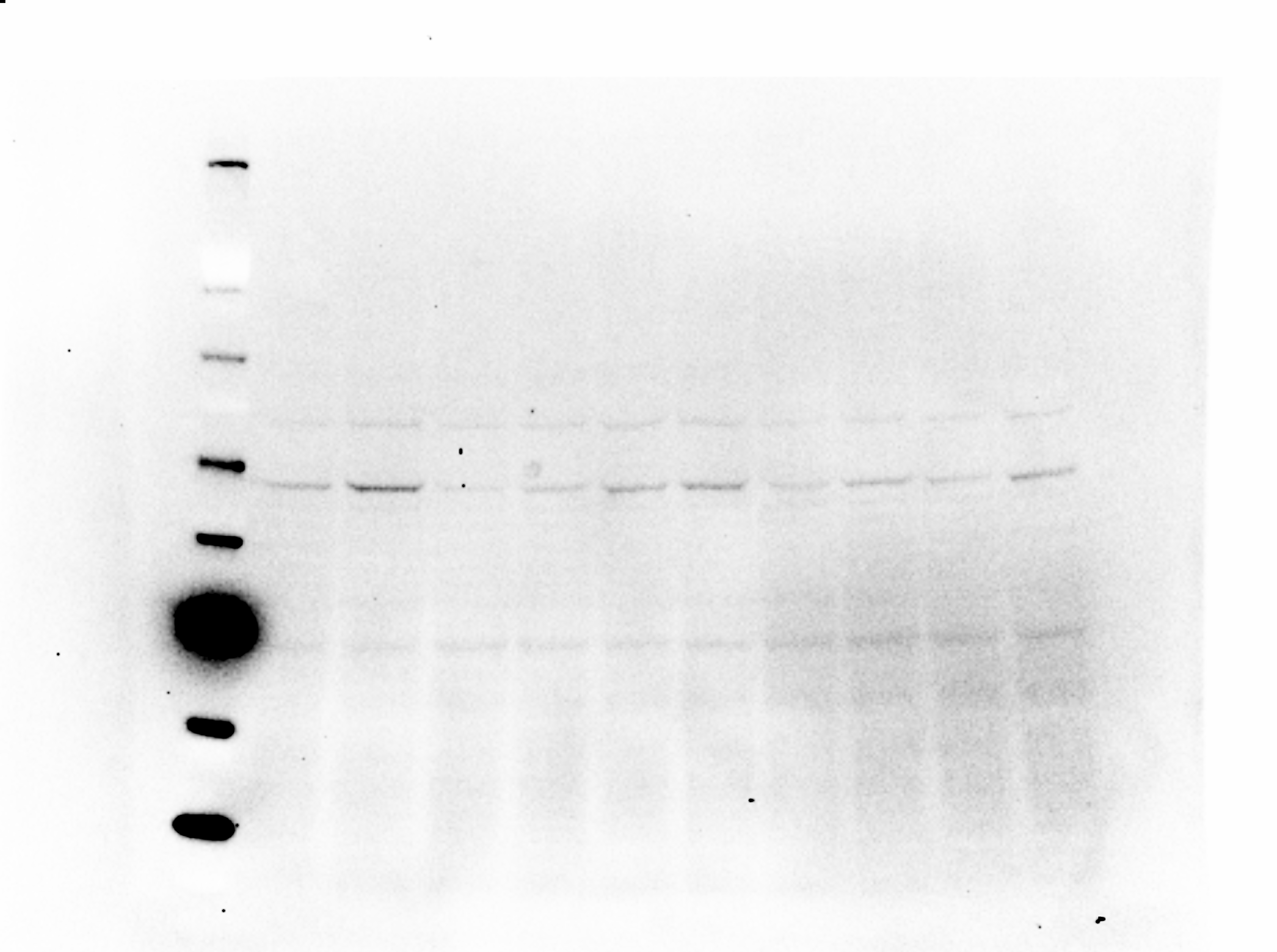

Supplement: Supplementary file 2 — Source Data [file 41467_2019_10811_MOESM2_ESM.zip › the Source Data file/Figure 4d/ALDH1A3_siRNAs with PIC-WB.jpg]

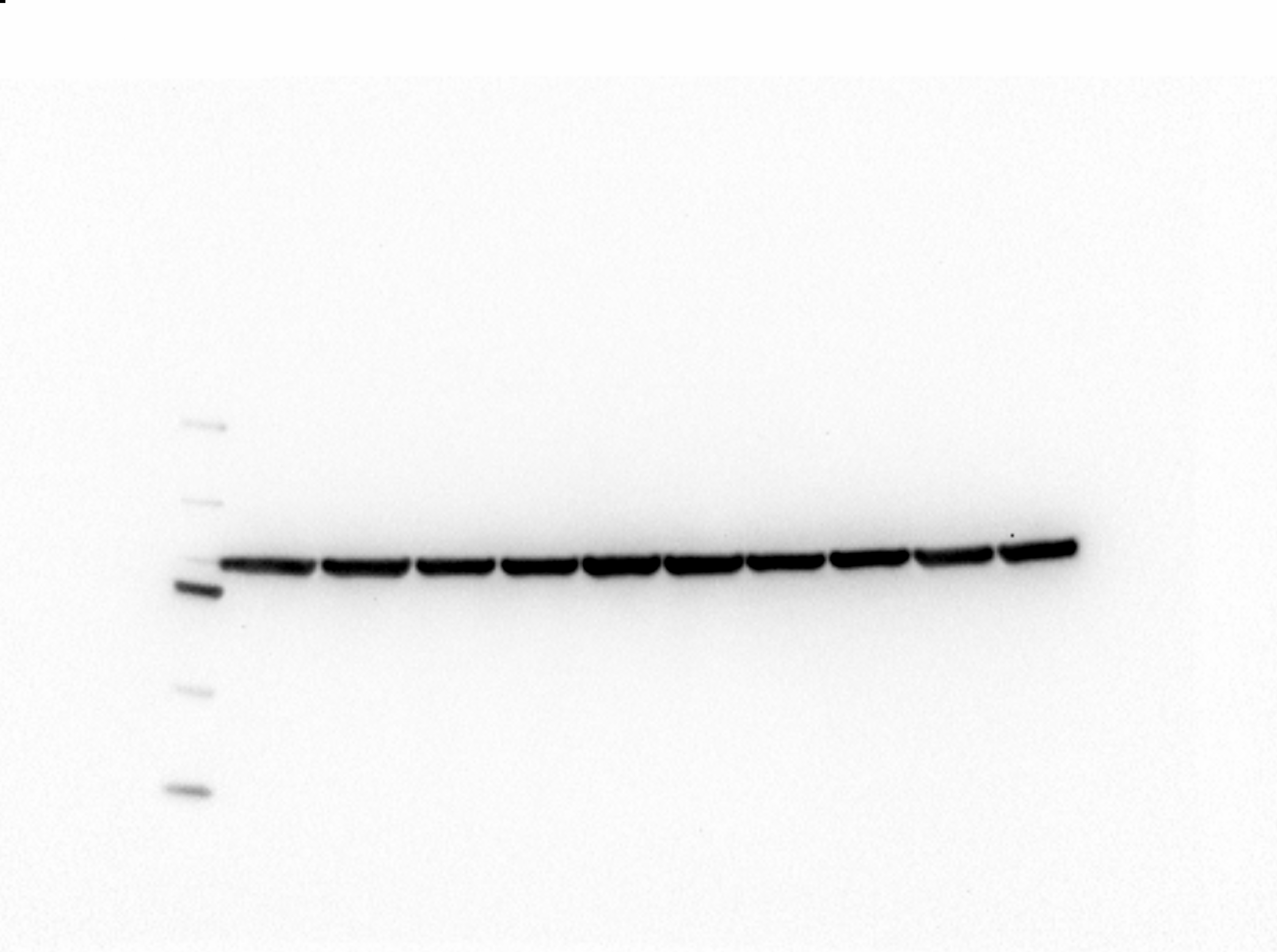

Supplement: Supplementary file 2 — Source Data [file 41467_2019_10811_MOESM2_ESM.zip › the Source Data file/Figure 4d/bActin for ALDH1A3_siRNAs with PIC-WB.jpg]

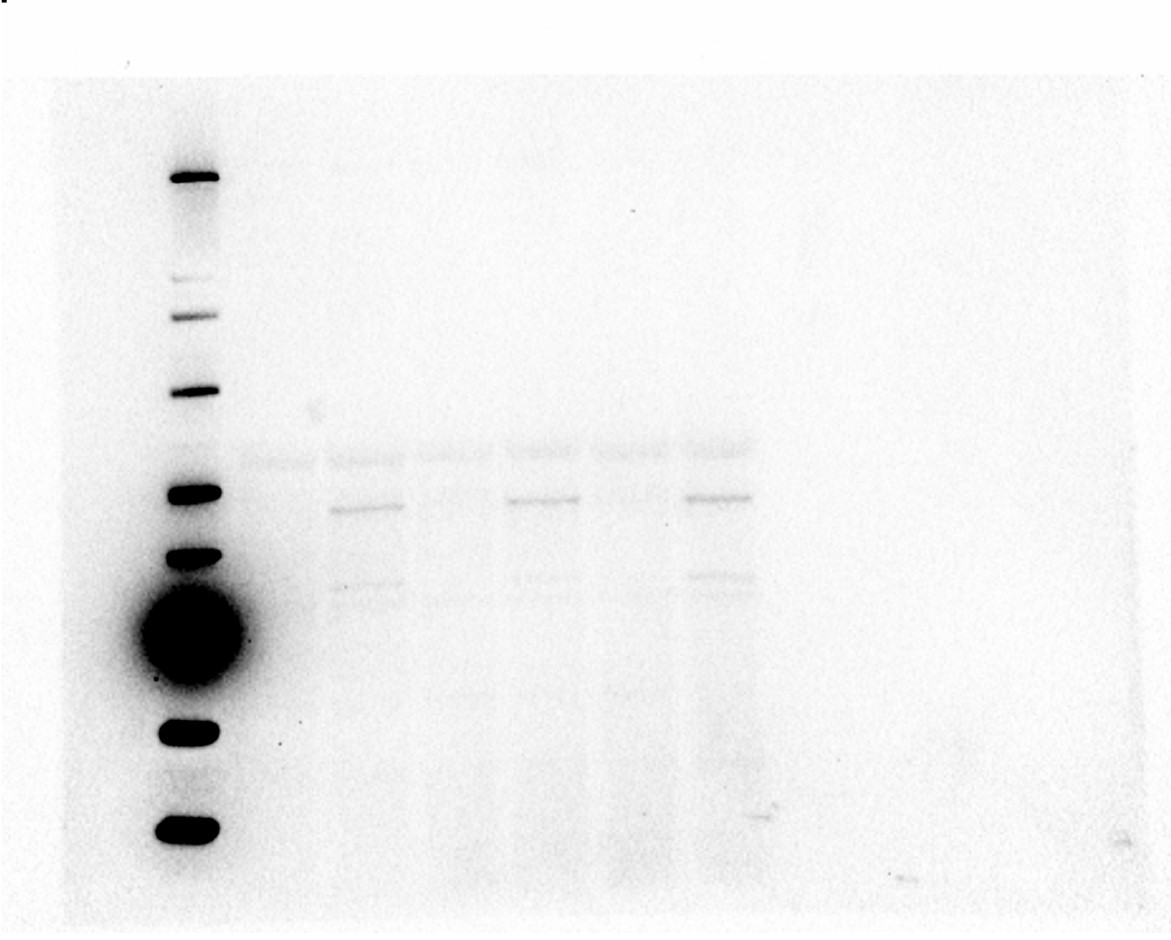

Supplement: Supplementary file 2 — Source Data [file 41467_2019_10811_MOESM2_ESM.zip › the Source Data file/Figure 4h/Aldh1a3 in center and edge-WB.jpg]

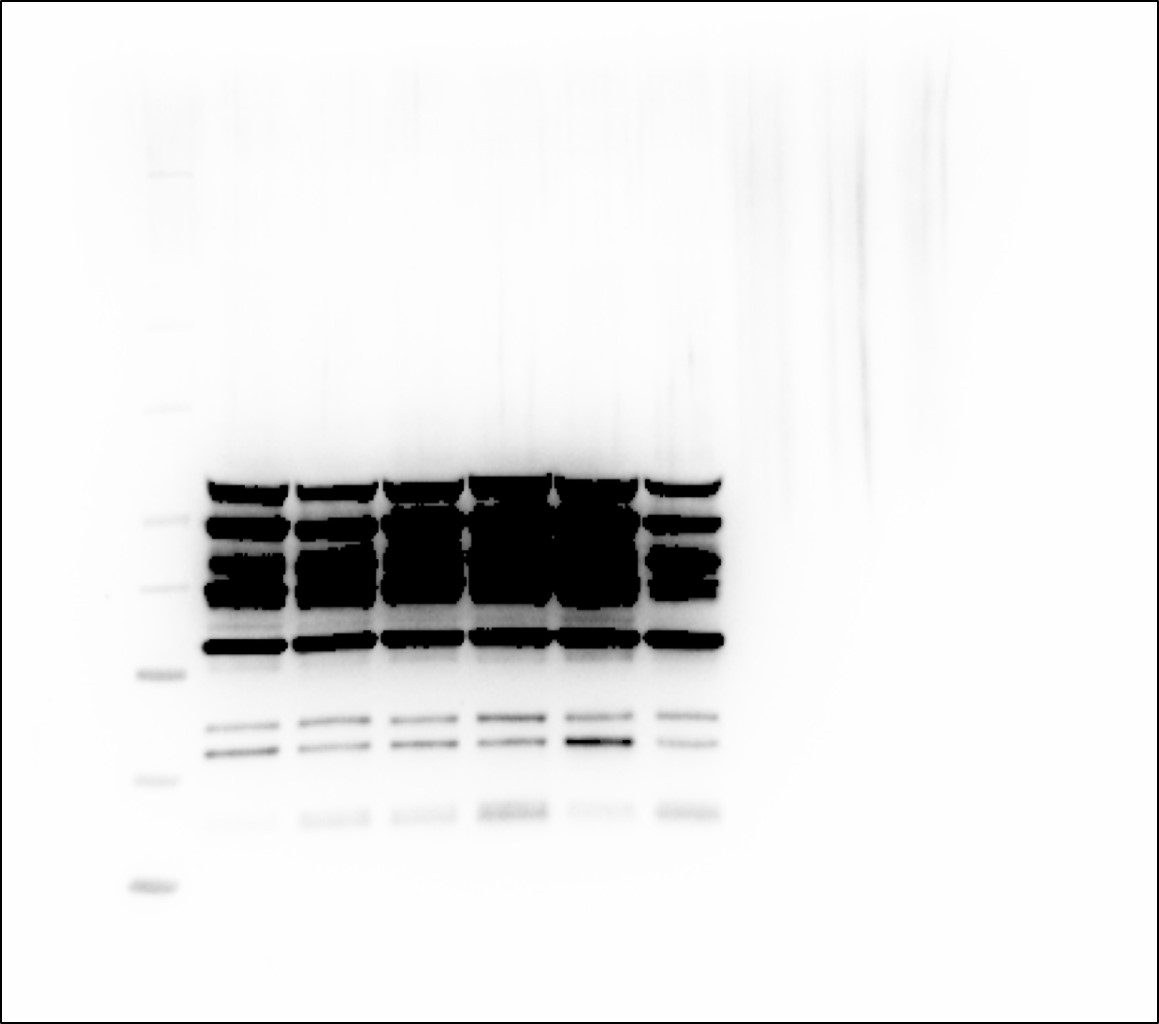

Supplement: Supplementary file 2 — Source Data [file 41467_2019_10811_MOESM2_ESM.zip › the Source Data file/Figure 4h/bActin in center and edge-WB.jpg]

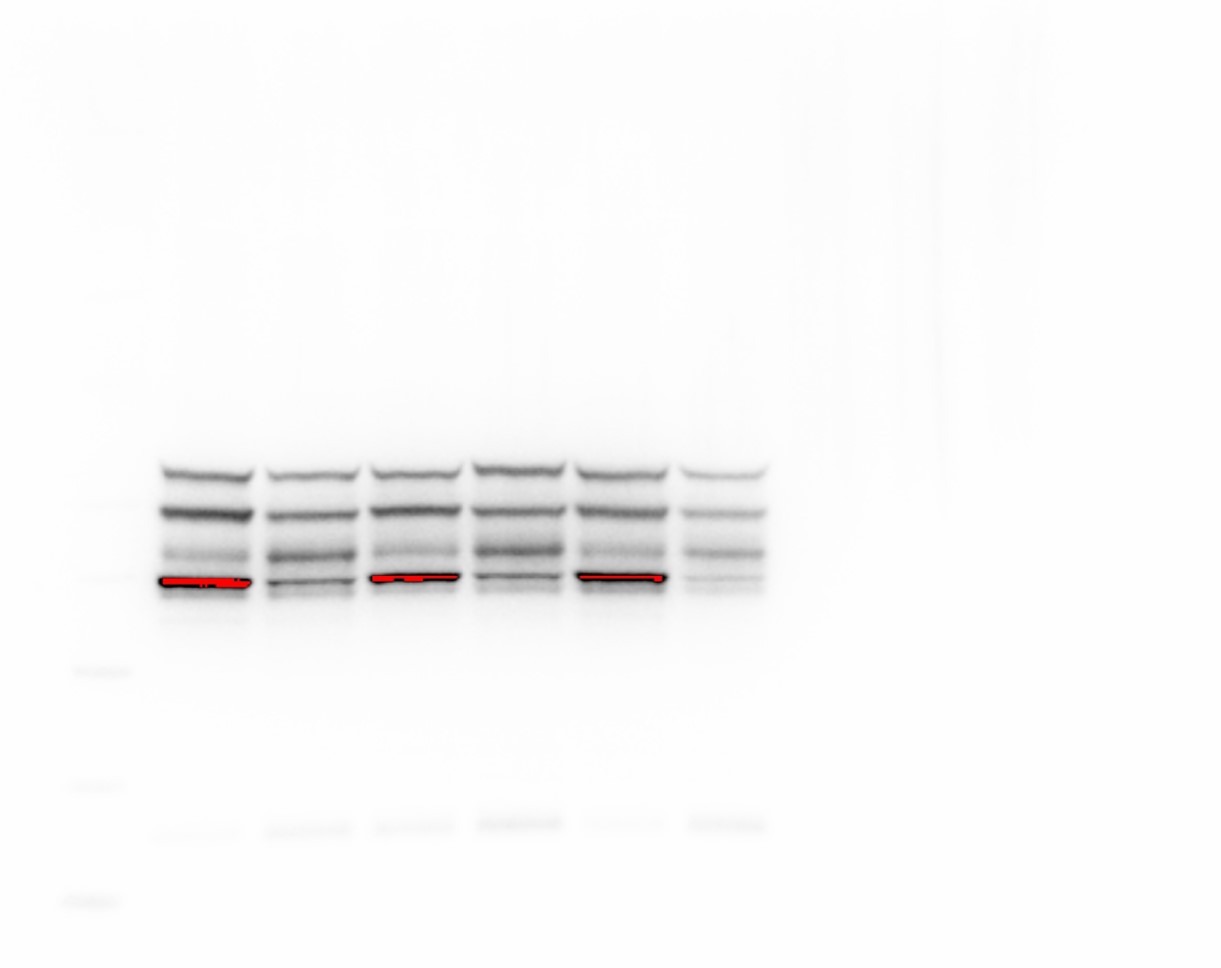

Supplement: Supplementary file 2 — Source Data [file 41467_2019_10811_MOESM2_ESM.zip › the Source Data file/Figure 4h/Krt15 in center and edge-WB.jpg]

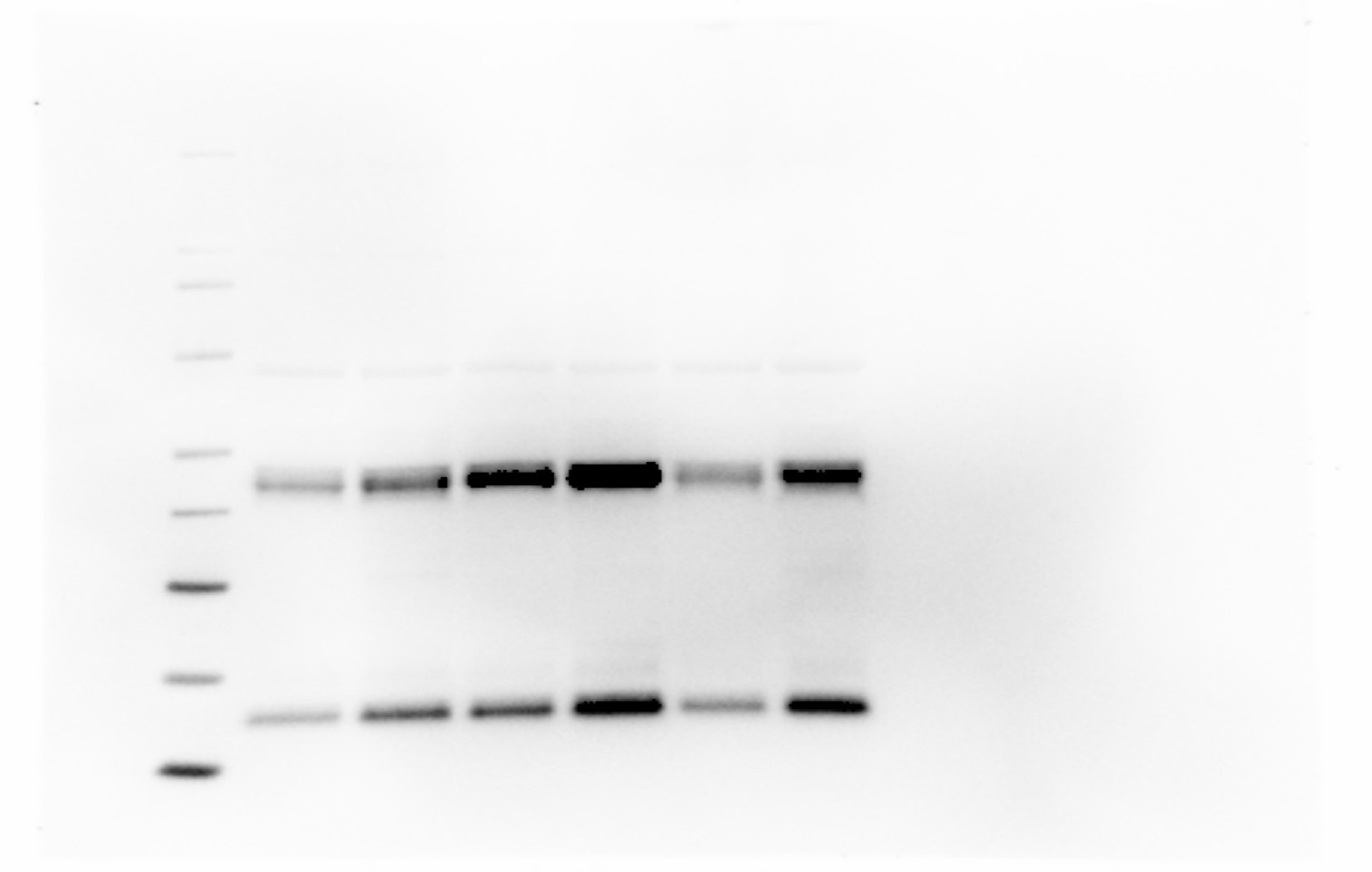

Supplement: Supplementary file 2 — Source Data [file 41467_2019_10811_MOESM2_ESM.zip › the Source Data file/Figure 4h/Krt19 in center and edge-WB.jpg]

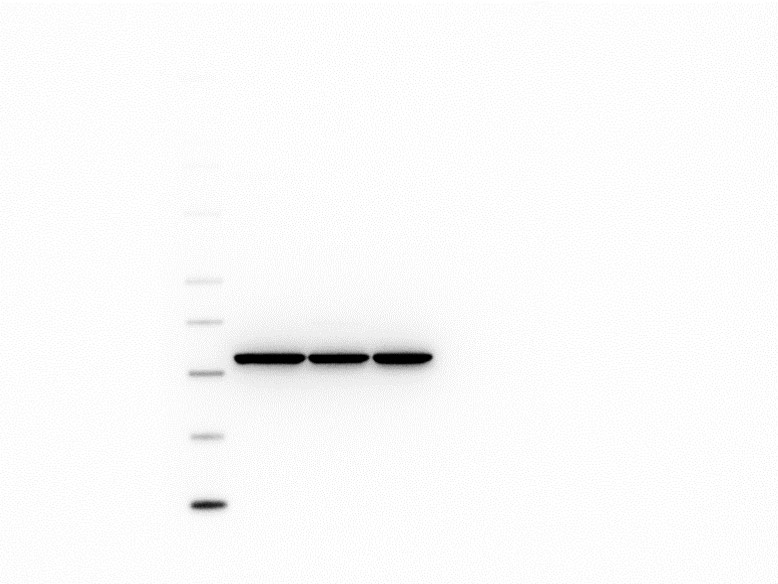

Supplement: Supplementary file 2 — Source Data [file 41467_2019_10811_MOESM2_ESM.zip › the Source Data file/Figure 5a/bActin_RA+BMS-WB.jpg]

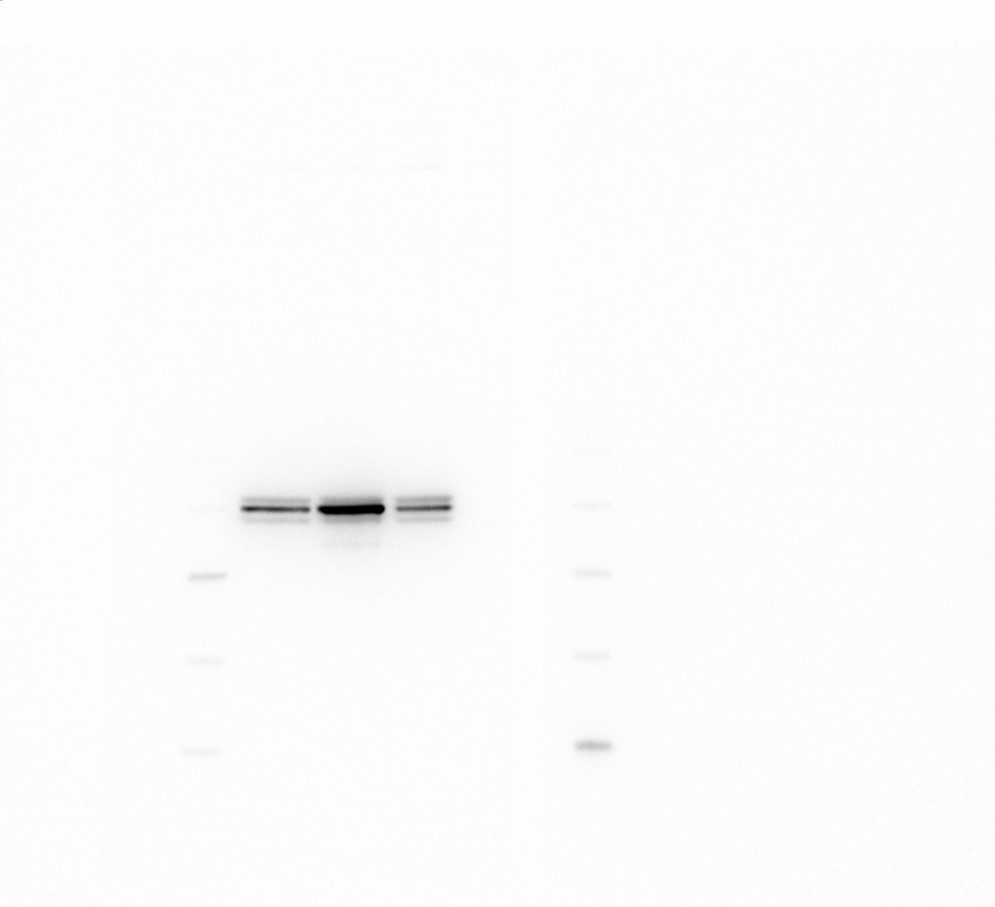

Supplement: Supplementary file 2 — Source Data [file 41467_2019_10811_MOESM2_ESM.zip › the Source Data file/Figure 5a/KRT15_RA+BMS-WB.jpg]

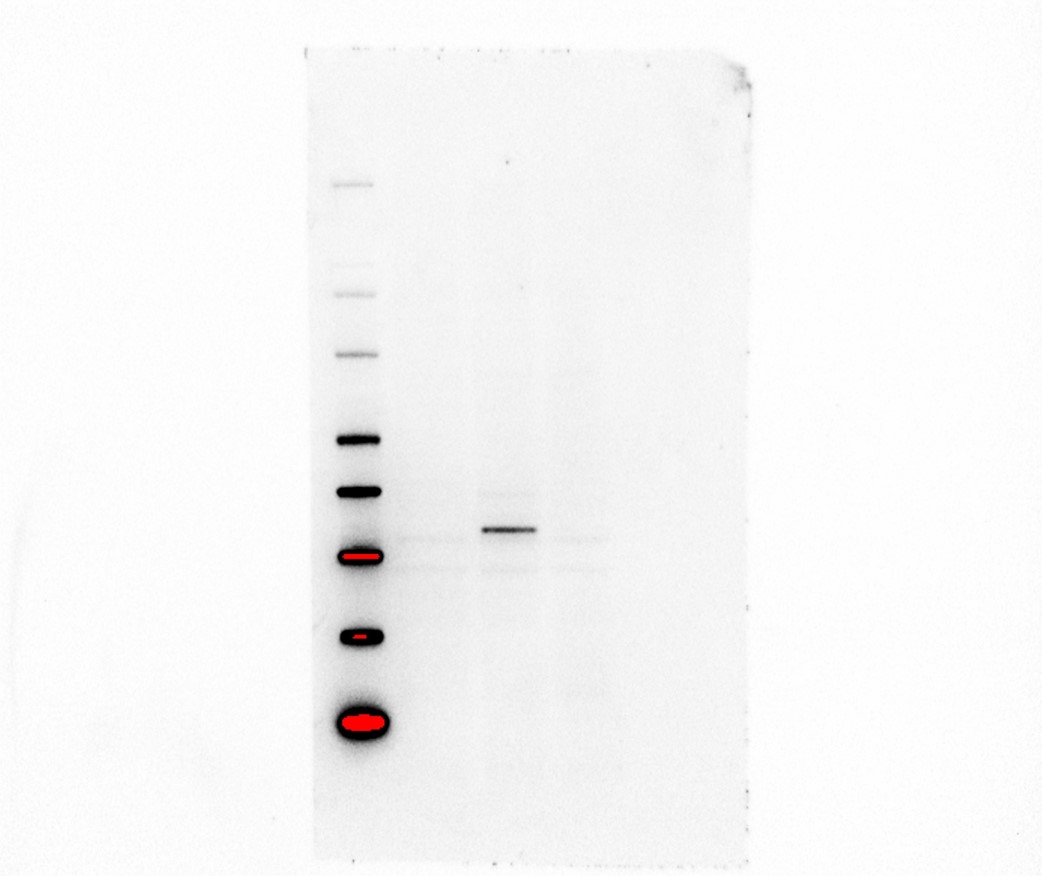

Supplement: Supplementary file 2 — Source Data [file 41467_2019_10811_MOESM2_ESM.zip › the Source Data file/Figure 5a/KRT19_RA+BMS-WB.jpg]

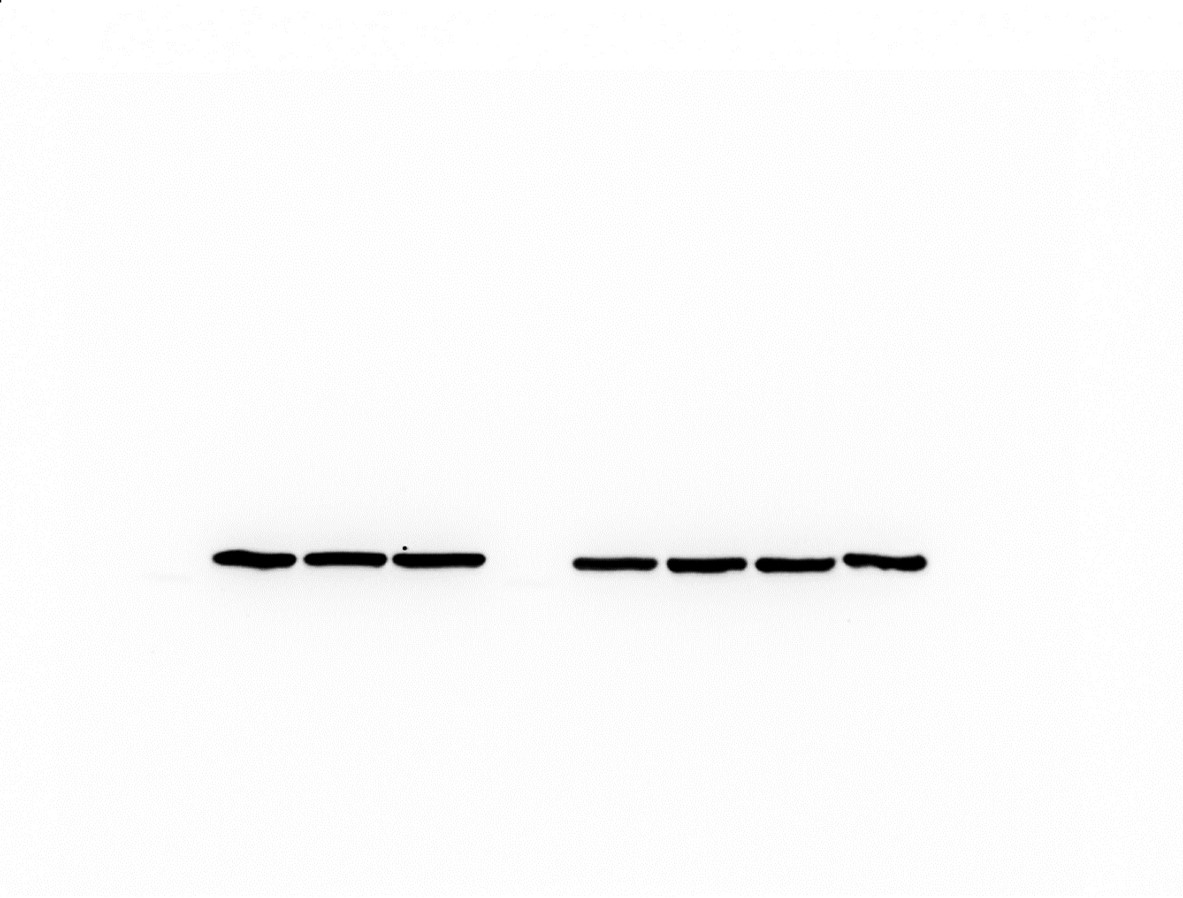

Supplement: Supplementary file 2 — Source Data [file 41467_2019_10811_MOESM2_ESM.zip › the Source Data file/Figure 5b/bActin_PIC+BMS-WB.jpg]

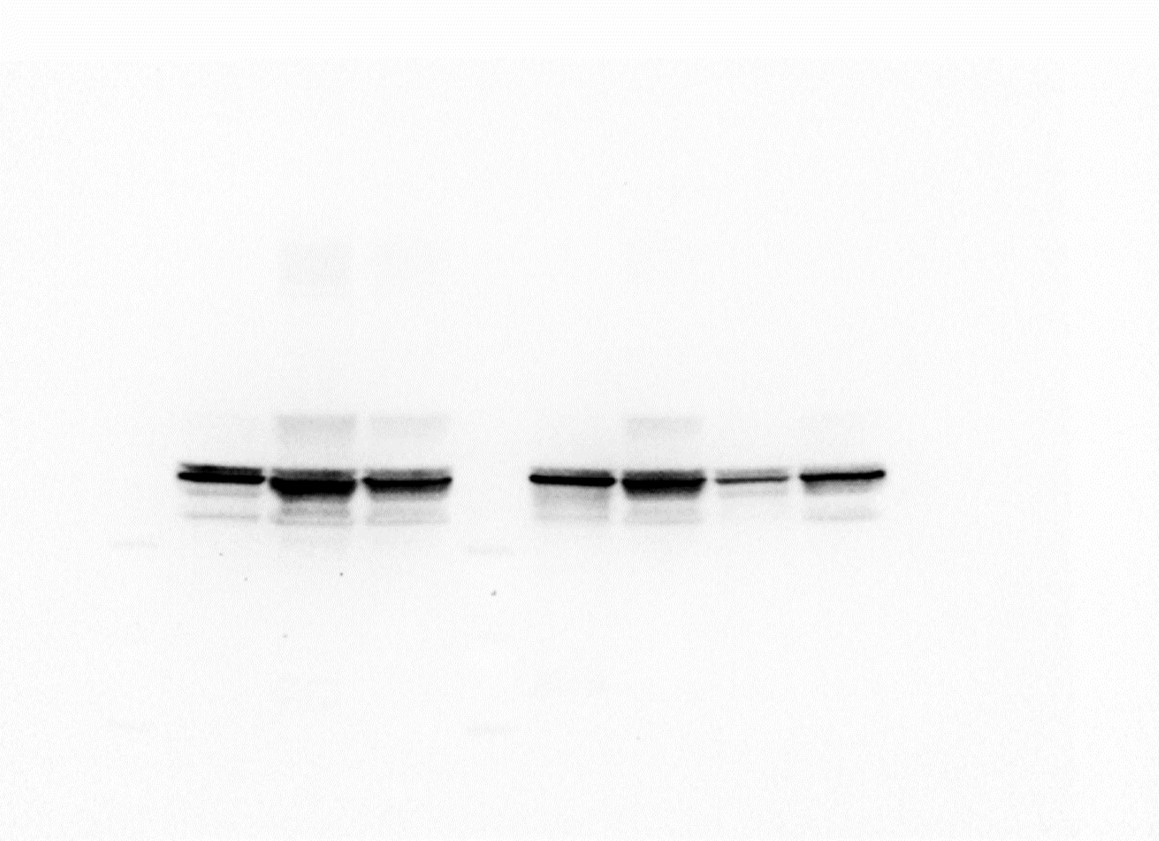

Supplement: Supplementary file 2 — Source Data [file 41467_2019_10811_MOESM2_ESM.zip › the Source Data file/Figure 5b/KRT15_PIC+BMS-WB.jpg]

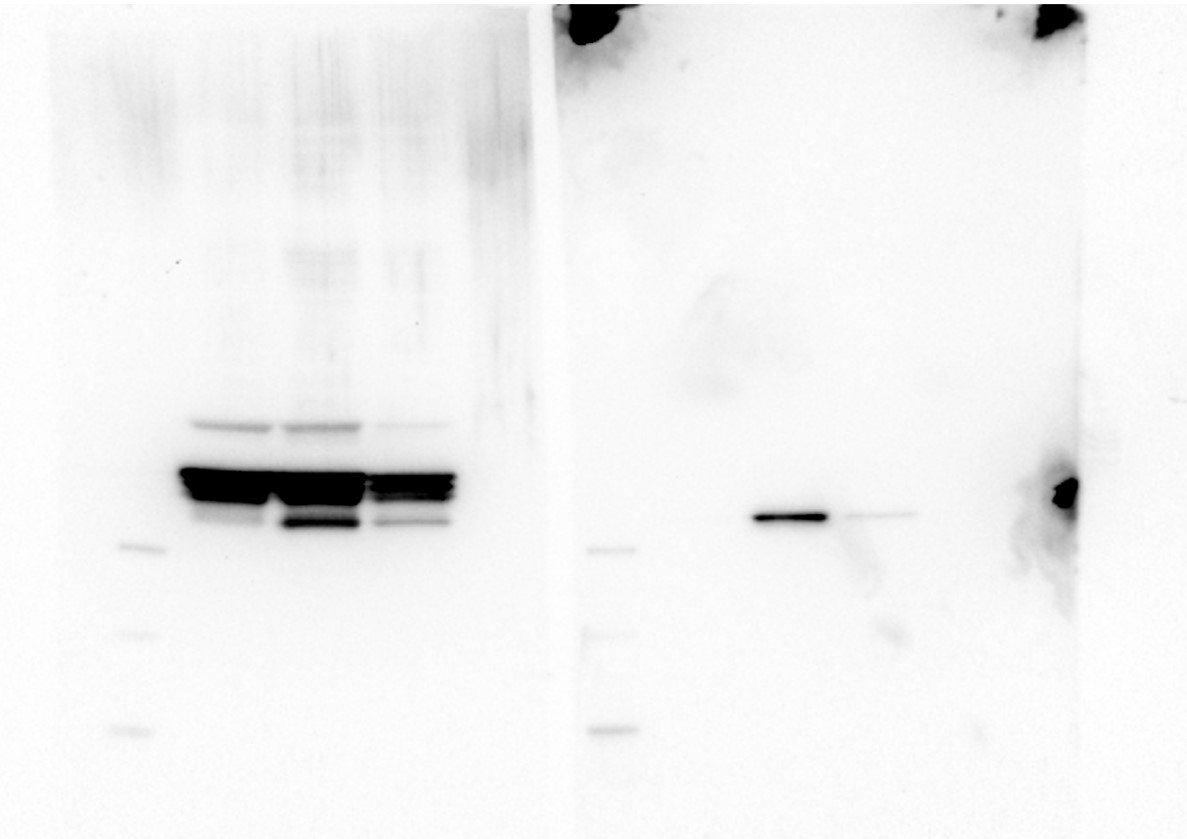

Supplement: Supplementary file 2 — Source Data [file 41467_2019_10811_MOESM2_ESM.zip › the Source Data file/Figure 5b/KRT19_PIC+BMS-WB.jpg]
